# Metabolic reprogramming of NK cells in chronic brucellar arthritis: role of the HIF-1α/mTOR axis

**DOI:** 10.3389/fimmu.2026.1961240

**Published:** 2026-10-06

**Authors:** Ganggang Sheng, Wenxuan Li, Da Liu, Xuanchen Ren, Mingyang Wei, Zijing Yang, Weiyu Tian, Fengbo Zhang, Yuejie Zhu, Fanxuan Zeng, Zhiqiang Zhao, Ping Xu, Hongyu Tian, Nannan Pang, Chaopeng Li, Zhendong Zhang

**Affiliations:** 1 Department of Orthopedic Surgery, The First Affiliated Hospital of Shihezi University, Shihezi, China; 2 Department of Clinical Laboratory, The First Affiliated Hospital of Xinjiang Medical University., Urumqi, Xinjiang, China; 3 Center for Translational Medicine, The First Affiliated Hospital of Shihezi University, Shihezi, Xinjiang, China; 4 Department of Pharmacy, The First Affiliated Hospital of Shihezi University, Shihezi, China

**Keywords:** brucellar arthritis, functional exhaustion, HIF−1α, metabolic reprogramming, mTOR, natural killer cells

## Abstract

**Background:**

Brucellar arthritis is the most common complication of brucellosis, and its chronic progression is closely associated with immune cell dysfunction. Natural killer (NK) cells play a central role in anti-infective immunity; however, their functional status and regulatory mechanisms in brucellar arthritis remain poorly understood.

**Methods:**

Peripheral blood and synovial tissue samples were collected from healthy controls, as well as from patients with acute-phase and chronic-phase brucellar arthritis. Single-cell RNA sequencing (scRNA-seq), flow cytometry, quantitative real-time PCR (qPCR), JC-1 mitochondrial membrane potential assay, Seahorse metabolic analysis, and Western blotting were employed to systematically evaluate NK cell phenotypes, functions, metabolic profiles, and HIF-1α/mTOR pathway activity. *In vitro*, heat-killed Brucella melitensis 16M (HKBA) was used to stimulate NK-92 cells and HIF-1α-knockout (KO) cell lines, with mTOR inhibitor intervention, to dissect the regulatory mechanism of the HIF-1α/mTOR axis in NK cell metabolic reprogramming.

**Results:**

scRNA-seq revealed enrichment of NK cells in the synovium of brucellar arthritis patients, with aberrant expression of oxidative phosphorylation (OXPHOS)-related genes. Clinical sample analyses showed that, compared with healthy controls, the proportion of peripheral blood NK cells increased in both acute- and chronic-phase patients; however, NK cells from chronic-phase patients exhibited significantly reduced IFN-γ, Granzyme B, and CD107a degranulation levels. HIF-1α protein was consistently overexpressed in patient NK cells, whereas phosphorylated mTOR-pathway proteins (p-PI3K, p-AKT, p-mTOR) were markedly suppressed. *In vitro*, HKBA treatment inhibited mTOR pathway activity in NK-92 cells in a dose- and time-dependent manner and reduced mitochondrial membrane potential as shown by JC-1 staining. HIF-1α knockout significantly restored NK cell cytotoxic function, upregulated OXPHOS- and glycolysis-related gene expression, and improved mitochondrial membrane potential. Transcriptomic sequencing confirmed enrichment of mTOR signaling and energy metabolism gene sets after knockout. Importantly, mTOR pathway inhibition completely blocked the HIF-1α-knockout-mediated restoration of NK cell function and mitochondrial fitness, indicating that HIF-1α primarily drives NK cell metabolic and functional abnormalities through mTOR suppression.

**Conclusions:**

Brucella infection induces HIF-1α overexpression, which suppresses mTOR signaling and drives metabolic reprogramming of NK cells characterized by impaired OXPHOS and functional exhaustion, thereby promoting chronicity of brucellar arthritis. Targeting the HIF-1α/mTOR axis may offer a novel therapeutic strategy for brucellar arthritis.

## 1 Introduction

Brucellosis is a widespread zoonotic infectious disease caused by bacteria of the genus Brucella (1, 2), with over 500,000 new human cases reported annually worldwide (2). It not only poses a serious threat to human health but also causes enormous economic losses to the livestock industry. Osteoarticular involvement is the most common complication of brucellosis, affecting approximately 40%–85% of patients, manifesting as arthritis, sacroiliitis, or spondylitis (3). In the acute phase (disease duration <3 months), brucellar arthritis typically presents with migratory severe pain in large joints—most commonly the knee, hip, and sacroiliac joints—with a stabbing nature directly linked to bacteremia and acute synovial inflammation (4). As the disease progresses to the chronic phase (>6 months), the core pathological features shift to bone destruction, joint erosion, and immune evasion, ultimately leading to joint dysfunction and even disability (5). Although combined antibiotic therapy effectively controls acute-phase infection, approximately 10%–30% of patients still develop chronic arthritis (6), largely due to our insufficient understanding of the persistent joint immunopathological damage triggered by Brucella infection.

Natural killer (NK) cells are key members of the innate immune system and serve as the first line of defense against infections. Without prior sensitization, NK cells directly kill pathogen-infected cells through perforin and granzyme release (7), while secreting cytokines such as IFN-γ to activate macrophages and adaptive immune responses (8). In acute Brucella infection, NK cells are rapidly mobilized to the infection site and, via IFN-γ production, activate macrophage bactericidal activity, which is critical for controlling early bacteremia. However, clinical evidence indicates that peripheral blood NK cell counts are reduced and functionally impaired in chronic brucellosis patients (9), with diminished IFN-γ and Granzyme B secretion, suggesting that Brucella may employ immune evasion mechanisms to undermine NK cell protection (10). To date, the key molecular mechanisms regulating NK cell functional status in brucellar arthritis remain undefined.

In recent years, metabolic reprogramming of immune cells has been recognized as a core event determining their functional fate. Resting NK cells primarily rely on mitochondrial oxidative phosphorylation (OXPHOS) for energy maintenance; upon activation, they rapidly switch to glycolysis (the Warburg effect) (11)to meet the demands for ATP and biosynthetic precursors required for rapid proliferation and effector functions (12). Flexible metabolic pathway switching is a prerequisite for normal NK cell anti-infective function (13). Hypoxia-inducible factor-1α (HIF-1α) is a pivotal transcription factor regulating metabolic switching. Under normoxia, it is ubiquitinated and degraded, whereas in hypoxic or inflammatory microenvironments, it stabilizes, translocates to the nucleus, and activates downstream glycolysis-related genes such as LDHA, PDK1, and GLUT1 (14, 15). Persistent HIF-1α overexpression in immune cells—including macrophages and T cells—remodels their metabolic phenotypes and influences inflammatory outcomes (16). However, the expression pattern of HIF-1α in NK cells from brucellar arthritis and its impact on metabolic pathways have not yet been reported.

Mammalian target of rapamycin (mTOR) is a central hub integrating energy metabolism, nutrient sensing, and immune signaling (17, 18). mTOR complex 1 (mTORC1) promotes protein synthesis, mitochondrial biogenesis, and glycolysis through phosphorylation of downstream effectors such as S6K and 4E-BP1, and is an essential signaling node for NK cell transition from resting to activated states (18). Studies show that sustained HIF-1α signaling in the tumor microenvironment can suppress mTOR activity via multiple mechanisms, including activation of the TSC1/2 complex and induction of REDD1, thereby restricting NK cell metabolism (19, 20). In chronic infection models, persistent antigen stimulation can drive NK cells into an “exhausted” phenotype similar to that of T cells (21), characterized by downregulated effector molecules, reduced metabolic activity, and mitochondrial dysfunction (22). However, whether HIF-1α induces NK cell metabolic reprogramming through mTOR inhibition to promote chronicity in brucellar arthritis remains unexplored.

Based on this background, we propose the hypothesis that in brucellar arthritis, Brucella infection induces aberrant HIF-1α overexpression in NK cells, which subsequently suppresses mTOR signaling, shifting NK cell energy metabolism from efficient mitochondrial OXPHOS to an inefficient and rigid glycolysis-dependent state. This leads to reduced mitochondrial respiratory reserve, insufficient ATP production, and impaired synthesis of effector molecules, ultimately driving NK cells toward functional inhibition and exacerbating Brucella immune evasion and chronic joint inflammation.

This study used heat inactivated Brucella 16M (HKBA) to stimulate NK cells, aiming to exclude confounding factors of live bacterial infection and separately analyze the direct effects of bacterial structural components on NK cell metabolism. Although Harms et al (23). pointed out that heat inactivated bacteria cannot induce arthritis in SKG mice, our use of HKBA is not to simulate complete infection, but to explore the initial metabolic events triggered by antigen recognition.

HKBA has the following unique advantages (1): eliminating metabolic hijacking - heat inactivated bacteria do not consume culture medium nutrients, avoiding the “pseudo starvation” state caused by competition between live bacteria and hosts for glucose and amino acids (24), ensuring true measurement of the nutrient sensitive pathway HIF-1 α/mTOR (2); Avoiding active intervention of virulence factors - live bacteria secrete effector proteins (such as BtpB) through T4SS to consume NAD ^+^/NADH/ATP and reprogram host metabolism (25). HKBA eliminates this active manipulation, allowing the observed metabolic changes to truly reflect the response of NK cells themselves (3); Accurately analyzing the initial signal driven by PAMPs - HKBA retains intact PAMPs such as lipoproteins and LPS, which is sufficient to trigger initial metabolic reprogramming through TLRs and avoid interference from complex downstream events of intracellular infections (26).

Therefore, HKBA is a controlled tool for studying the direct drive of NK cell metabolic reprogramming by Brucella antigens, particularly suitable for the antigen rich joint microenvironment during chronic arthritis. We acknowledge its limitations and emphasize the need to validate the conclusions of this study in the context of live bacterial infections in the future.

The aims of this study are (1): to systematically characterize the phenotype, function, and metabolic features of NK cells in peripheral blood and synovium of brucellar arthritis patients (2); to define the critical role of the HIF-1α/mTOR signaling axis in Brucella-induced NK cell metabolic reprogramming; and (3) to validate the potential of targeting this axis as a strategy to reverse NK cell suppression and block chronicity, using HIF-1α gene knockout and mTOR inhibitor intervention. This study will provide new insights into the immunopathological mechanisms of brucellar arthritis and offer a theoretical basis for developing metabolism-based immunotherapies.

## 2 Materials and methods

### 2.1 Study subjects

This study enrolled patients with brucellar arthritis and healthy controls who visited The First Affiliated Hospital of Shihezi University between July 2024 and May 2026.Subjects were divided into three groups: acute-phase brucellar arthritis (A-BA, n=20), chronic-phase brucellar arthritis (C-BA, n=20), and healthy controls (HC, n=20). Healthy controls were age- and sex-matched individuals without a history of Brucella infection or immune-related diseases. All participants provided written informed consent. The study was approved by the Ethics Committee of The First Affiliated Hospital of Shihezi University (ethics approval no. KJ2025-640-01).

In addition, experiments involving live Brucella were conducted in the BSL-3 laboratory of the Xinjiang Center for Disease Control and Prevention (Xinjiang CDC). This facility complies with national biosafety standards CNAS-CL05:2009 and GB 19489:2008 and holds BSL-3 operational certification from the China National Accreditation Service for Conformity Assessment (CNAS, registration no. CNAS BL0099). All operators followed BSL-3 protocols, performed Brucella culture and preparation in A2-type biological safety cabinets, and wore full personal protective equipment. It should be noted that all *in vitro* functional experiments in this study used heat-killed Brucella melitensis 16M (HKBA) and did not involve live bacterial infection of cells; nevertheless, all related operations were conducted according to the above biosafety standards to ensure safety.

### 2.2 Inclusion and exclusion criteria

Inclusion criteria: Patients fulfilling the Chinese Ministry of Health diagnostic criteria for brucellosis and meeting all of the following (1): serum agglutination test (SAT) titer ≥1:100++ (2); complement fixation test (CFT) titer ≥1:10++ (3); anti-human immunoglobulin test (Coombs) titer ≥1:400++; and (4) typical osteoarticular changes on X-ray, CT, or MRI (bone destruction, joint swelling, soft tissue swelling, etc.) (27, 28).Staging criteria: Acute phase was defined as osteoarticular symptoms appearing within 3 months of infection; chronic phase was defined as persistent osteoarticular damage lasting >6 months after infection.

Exclusion criteria (1): diabetes, malignancy, or severe cardiac, hepatic, or renal insufficiency (2); rheumatic immune diseases within the past 5 years, or use of immunosuppressants or glucocorticoids within 1 month (3); other infectious diseases (tuberculosis, typhoid, HIV, etc.) (4); severe joint deformity or prior joint replacement (5); pregnancy or lactation.

### 2.3 Peripheral blood mononuclear cell isolation

Fasting venous blood (10 mL) was collected in EDTA-anticoagulated tubes. PBMCs were isolated by Ficoll-Paque PLUS (GE Healthcare, USA) density gradient centrifugation at 2000 rpm for 20 min. The buffy coat layer was collected, washed twice with PBS, and resuspended in RPMI 1640 medium supplemented with 10% fetal bovine serum (FBS, Gibco, USA) for subsequent flow cytometry and functional assays.

### 2.4 Flow cytometry

Surface staining: 1×10^6^ PBMCs were blocked with human Fc receptor blocking reagent for 10 min at room temperature, followed by incubation with the following fluorescently conjugated primary antibodies (all at 1:100 dilution): CD3-FITC (clone UCHT1, cat. 555332, BD Pharmingen), CD56-APC (clone B159, cat. 555518, BD Pharmingen), and CD107a-PE (clone H4A3, cat. 555801, BD Pharmingen) at 4 °C in the dark for 30 min. After washing twice with PBS, cells were stained with Fixable Viability Dye 777 (cat. S0D0026-100T, STARTER) for 15 min at room temperature in the dark, washed, and resuspended for acquisition.

For intracellular IFN-γ detection, PBMCs were first stimulated with PMA (50 ng/mL), ionomycin (1 μg/mL), and brefeldin A for 4 h, then fixed and permeabilized using the Cytofix/Cytoperm kit (cat. 554714, BD Pharmingen) according to the manufacturer’s instructions, followed by intracellular staining with IFN-γ-PE-Cy7 (clone B27, cat. 557643, BD Pharmingen) at 4 °C in the dark for 30 min, washed, and resuspended.

All samples were acquired on a BD FACSLyric flow cytometer, and data were analyzed with FlowJo v10.8. Debris and dead cells were excluded by FSC-A/SSC-A and viability dye gating. NK cells were defined as CD3^-^CD56^+^, and the percentages of CD107a^+^ and IFN-γ^+^ cells were determined.

Mitochondrial membrane potential (JC-1) assay: 1×10^5^ NK-92 cells were incubated with 2 μmol/L JC-1 probe at 37 °C in the dark for 20 min, washed three times with pre-chilled PBS, seeded onto slides, fixed with paraformaldehyde, and imaged under a fluorescence microscope for red (JC-1 aggregates) and green (JC-1 monomers) fluorescence. ImageJ software was used to quantify fluorescence intensity, and the red/green ratio was calculated.

### 2.5 Cytokine detection

Serum concentrations of IFN-γ, TNF-α, Granzyme B, IL-10, IL-13, and IL-18 were measured using ELISA kits (all from JONLNBIO, China) according to the manufacturer’s instructions.

### 2.6 Western blotting

Protein extraction: NK-92 cells or PBMCs were lysed in RIPA buffer containing protease and phosphatase inhibitors (Thermo Fisher, USA) on ice for 30 min, centrifuged at 12,000×g for 10 min at 4 °C, and the supernatant was collected. Protein concentration was determined by BCA assay (Pierce™, USA).

Electrophoresis and transfer: 20 μg of protein was separated by 10% SDS-PAGE and transferred to PVDF membranes (Immobilon-P, Millipore, USA). Membranes were blocked with 5% non-fat milk for 1 h at room temperature.

Antibody incubation: Primary antibodies were incubated overnight at 4 °C, including: HIF-1α (Abcam, ab228649, 1:1000), total PI3K p85β (Proteintech, 83606-5-RR, 1:1000), p-PI3K p85α (Invitrogen, MA5-41128, 1:1000), total AKT (CST #4691, 1:1000), p-AKT Ser473 (CST #4060, 1:2000), total mTOR (Proteintech, 28273-1-AP, 1:1000), p-mTOR Ser2448 (Proteintech, 80596-1-RR, 1:1000), total S6 (Proteintech, 66886-1-Ig, 1:1000), p-S6 Ser235 (Proteintech, 67898-1-Ig, 1:1000), total p70S6K (Proteintech, 14485-1-AP, 1:1000), p-p70S6K Thr389 (Proteintech, 28735-1-AP, 1:1000), Granzyme B (Proteintech, 13588-1-AP, 1:1000), and β-Actin (Proteintech, 81115-1-RR, 1:5000). After washing three times with TBST, HRP-conjugated secondary antibodies (ZSGB-BIO ZB-2305, 1:5000) were incubated for 1 h at room temperature. Signals were visualized by ECL (Thermo Fisher) and quantified using ImageJ.

### 2.7 Immunohistochemistry

Take paraffin embedded specimens of synovial tissue from the pathology department of the First Affiliated Hospital of Shihezi University, and slice them continuously with a thickness of 4 μ m. After dewaxing with xylene and gradient ethanol hydration, the slices were placed in sodium citrate buffer (pH 6.0) and subjected to antigen thermal repair using microwave repair method (95 °C, 15 min). After natural cooling to room temperature, incubate with 3% hydrogen peroxide for 10 minutes to block endogenous peroxidase activity; Subsequently, block with 5% goat serum at room temperature for 30 minutes. Add rabbit anti human HIF-1 α monoclonal antibody (Abcam, ab228649, dilution ratio 1:200) and mouse anti human Granzyme B monoclonal antibody (Proteintech, 13588-1-AP, dilution ratio 1:200) separately, and incubate overnight at 4 °C in a wet box. The next day, after washing with PBS three times, horseradish peroxidase (HRP) labeled goat anti rabbit/anti mouse universal secondary antibody (ZSGB-BIO, dilution ratio 1:500) was added dropwise and incubated at room temperature for 1 hour. After washing with PBS, DAB staining solution (Zhongshan Jinqiao) was used to avoid light and the staining time was controlled under a microscope until a positive brown signal appeared and the background was clear. Distilled water was used to terminate the staining. Hematoxylin counterstain cell nuclei for 1 minute, hydrochloric acid alcohol differentiation, ammonia water return to blue, gradient ethanol dehydration, xylene transparency, neutral gum sealing. Replace the primary antibody with PBS as the negative control. Synovial tissue specimens were obtained from 3 healthy controls and 3 patients with chronic phase brucellosis arthritis. Acute phase specimens were not obtained due to the risk of bacteremia phase biopsy. Randomly select 5 high-power fields (× 400) from each slice, analyze them using ImageJ software, and calculate the percentage of positive staining area (Positive Area%).

### 2.8 Cell culture and treatments

The NK-92 human natural killer cell line (cat. CL-0530) was purchased from Procell and authenticated by STR profiling. Complete culture medium consisted of MEMα (PM150422) supplemented with 0.2 mM inositol, 0.1 mM β-mercaptoethanol, 0.02 mM folic acid, 12.5% horse serum (HS, 164215), 12.5% fetal bovine serum (FBS, 164210), and 1% penicillin/streptomycin (P/S, PB180120). Recombinant human IL-2 (PB180634) was added before use. Cells were maintained at 37 °C in 5% CO_2_ and passaged every 2–3 days.

Preparation of heat-killed Brucella melitensis 16M (HKBA): B. melitensis 16M (ATCC 23456) was obtained from the China Institute of Veterinary Drug Control (IVDC; Beijing, China). The strain was cultured on Brucella agar at 37 °C with 5% CO_2_ for 72 h. Colonies were collected, washed three times with sterile PBS, and resuspended. The suspension was heat-inactivated at 70 °C for 30 min, verified by plating for no growth, aliquoted, and stored at −70 °C. Before use, the concentration was adjusted to 1×10¹¹ CFU/mL with sterile PBS (29).

HKBA treatment: NK-92 cells were co-cultured with HKBA at different multiplicities of infection (MOI): 0 (control), 1, 10, 50, 100, 200, and 500 (30, 31), for 24, 48, or 72 h. In some experiments, cells were pretreated with the mTOR inhibitor rapamycin (100 nM, MCE) for 1 h before HKBA stimulation.

### 2.9 CRISPR/Cas9 construction of HIF-1α-knockout (KO) NK-92 cell line

The human HIF1A homozygous knockout NK-92 cell line (clone 1D9) was generated by GenePharma using RNP electroporation-mediated large genomic deletion. The target gene NCBI Gene ID is 3091, transcript NM_181054.3.

gRNA design: Dual cleavage sites upstream of exon 2 and downstream of exon 10 of HIF1A were selected to delete the 18.5 kb coding region. Synthetic sgRNA sequences:

gRNA-A1: 5′-AGTTTCTCTAGCCTTAATCG-3′;

gRNA-A2: 5′-CATGTGCTCAGGTCTAGGTT-3′.

Transfection and selection: NK-92 cells were electroporated using the Amaxa Nucleofector™ (Lonza, Switzerland). At 48 h post-transfection, puromycin (1 μg/mL) was added for 7 days of selection. Monoclonal selection was performed by limiting dilution, and genomic PCR of the target region followed by sequencing confirmed frameshift mutations. KO cells were stably maintained in IL-2-containing medium.

### 2.10 NK-92 cell effector function assay

WT or HIF-1α KO NK-92 cells were co-cultured with K562 target cells or stimulated with HKBA (MOI=100). Medium alone and PMA/Ionomycin were used as negative and positive controls, respectively. After incubation, cells were harvested and stained for surface markers (CD56), followed by fixation and permeabilization using a Cytofix/Cytoperm kit for intracellular IFN-γ staining. Samples were acquired on a flow cytometer, and the percentages of CD107a^+^ and IFN-γ^+^ cells within the NK-92 (CD56⁺) population were analyzed using FlowJo.

### 2.11 Seahorse XFe96 metabolic analysis

Cell preparation: NK-92 cells treated with HKBA (MOI = 100) for 72 h were seeded at 2×10^5^ cells/well into XFe96 cell culture plates (Seahorse Bioscience, USA) and equilibrated for 1 h at 37 °C in a CO_2_-free incubator.

Mitochondrial stress test: Oligomycin (1 μmol/L), FCCP (1 μmol/L), and rotenone/antimycin A (0.5 μmol/L) were sequentially injected to measure basal oxygen consumption rate (OCR), maximal respiration, ATP-linked respiration, and spare respiratory capacity (SRC).

Glycolysis stress test: Glucose (10 mmol/L), oligomycin (1 μmol/L), and 2-deoxy-D-glucose (50 mmol/L) were sequentially injected to measure extracellular acidification rate (ECAR), basal glycolysis, and glycolytic capacity. All data were acquired with Wave software (Seahorse) and normalized to protein content.

### 2.12 Single-cell RNA sequencing

Healthy control synovial tissue was obtained from patients undergoing arthroscopic meniscectomy or diagnostic arthroscopy for traumatic meniscal tears, with no history of inflammatory joint disease or Brucella infection. Selection criteria included absence of synovitis upon macroscopic inspection and normal histology.

Acute-phase patients were not included for synovial biopsy because they were in an active bacteremic state, which poses an elevated risk of bleeding and dissemination of infection during invasive procedures, making such biopsies clinically unsafe and ethically unjustifiable. Therefore, acute-phase synovial tissues were not available for this study.

Tissue dissociation: Synovial tissue samples from healthy controls (n=3) and chronic-phase patients (n=4) were minced and digested with collagenase IV (1 mg/mL, Sigma) and DNase I (0.1 mg/mL, Roche) at 37 °C for 30 min, then filtered through a 70 μm cell strainer to obtain single-cell suspensions. For scRNA-seq, each sample was processed individually (not pooled) to preserve inter-subject variability. Library preparation was performed separately for each subject.

Library construction and sequencing: Approximately 10,000 cells per sample were captured using the 10x Genomics Chromium system (Next GEM chip), and single-cell transcriptome libraries were constructed with the Chromium Single Cell 3′ v3.1 kit. Sequencing was performed on an Illumina NovaSeq 6000 (PE150) at a depth of ~50,000 reads per cell.

Data analysis: Cell Ranger 6.0 was used for alignment (reference genome GRCh38) and expression matrix generation. Seurat 4.0 was used for quality control (filtering cells with <200 or >6,000 genes and >20% mitochondrial reads), principal component analysis, and UMAP dimensional reduction clustering. Differentially expressed genes (log2FC > 0.5, padj < 0.05) were subjected to KEGG enrichment and gene set enrichment analysis (GSEA, MSigDB Hallmark gene sets).

### 2.13 Transcriptome sequencing (RNA-seq)

WT and HIF-1α KO NK-92 cells were treated with HKBA (MOI = 100) for 72 h, with three biological replicates per group. Total RNA was extracted with Trizol (Invitrogen), and RIN ≥ 8 was confirmed on an Agilent 2100 Bioanalyzer. Libraries were constructed using the TruSeq Stranded mRNA Library Prep Kit (Illumina) and sequenced on a NovaSeq 6000 (PE150). Data were quality-controlled with fastp, aligned to the reference genome with STAR, and quantified with featureCounts. DESeq2 was used to identify differentially expressed genes (|log2FC| > 1, padj < 0.05). KEGG and GO analyses were performed using clusterProfiler (R package). The RNA-seq data have been deposited under accession number HRA020744.

### 2.14 Statistical analysis

Sample size determination: The sample size of 20 patients per clinical group (HC, A-BA, and C-BA) was determined based on effect size estimates from the single-cell immunophenotyping study of brucellosis by Wang et al. (ref (23).), which reported NK cell effector metrics. With a two-sided significance level (α) of 0.05 and a statistical power (1-β) of 0.80, a sample size of 20 per group was calculated to detect a clinically meaningful difference of approximately 30% in the percentage of IFN-γ-producing NK cells between groups. Considering potential sample loss, 20 patients were enrolled per group. Statistical analyses were performed using GraphPad Prism 10.0. All data are presented as mean ± SD. Normality was assessed using the Shapiro-Wilk test before choosing parametric versus non-parametric tests. Data with normal distribution were analyzed using independent-samples t-test (two groups) or one-way ANOVA followed by Tukey’s post-hoc test (multiple groups). Non-normally distributed data were analyzed using the Mann-Whitney U test or Kruskal-Wallis test. For multiple comparisons, P values were adjusted using the Benjamini-Hochberg false discovery rate method where applicable. Correlations were analyzed by Spearman’s rank correlation. P < 0.05 was considered statistically significant. All experiments were independently repeated at least three times.

## 3 Results

### 3.1 NK cells are enriched in the synovium of brucellar arthritis patients and exhibit metabolic abnormalities

To characterize the local immune microenvironment in brucellar arthritis, we performed single-cell RNA sequencing (scRNA-seq) on synovial tissue samples from chronic-phase brucellar arthritis patients and healthy controls using the 10x Genomics platform. The experimental workflow included synovial tissue biopsy, tissue dissociation, GEM formation and barcoding, followed by single-cell data analysis and cell clustering (**Figure 1A**). Uniform manifold approximation and projection (UMAP) and t-distributed stochastic neighbor embedding (t-SNE) algorithms were employed for dimensionality reduction and visualization of all cells; each dot represents a single cell, and cell types were annotated based on unsupervised clustering and canonical marker expression (**Figure 1B**).

**Figure 1:**
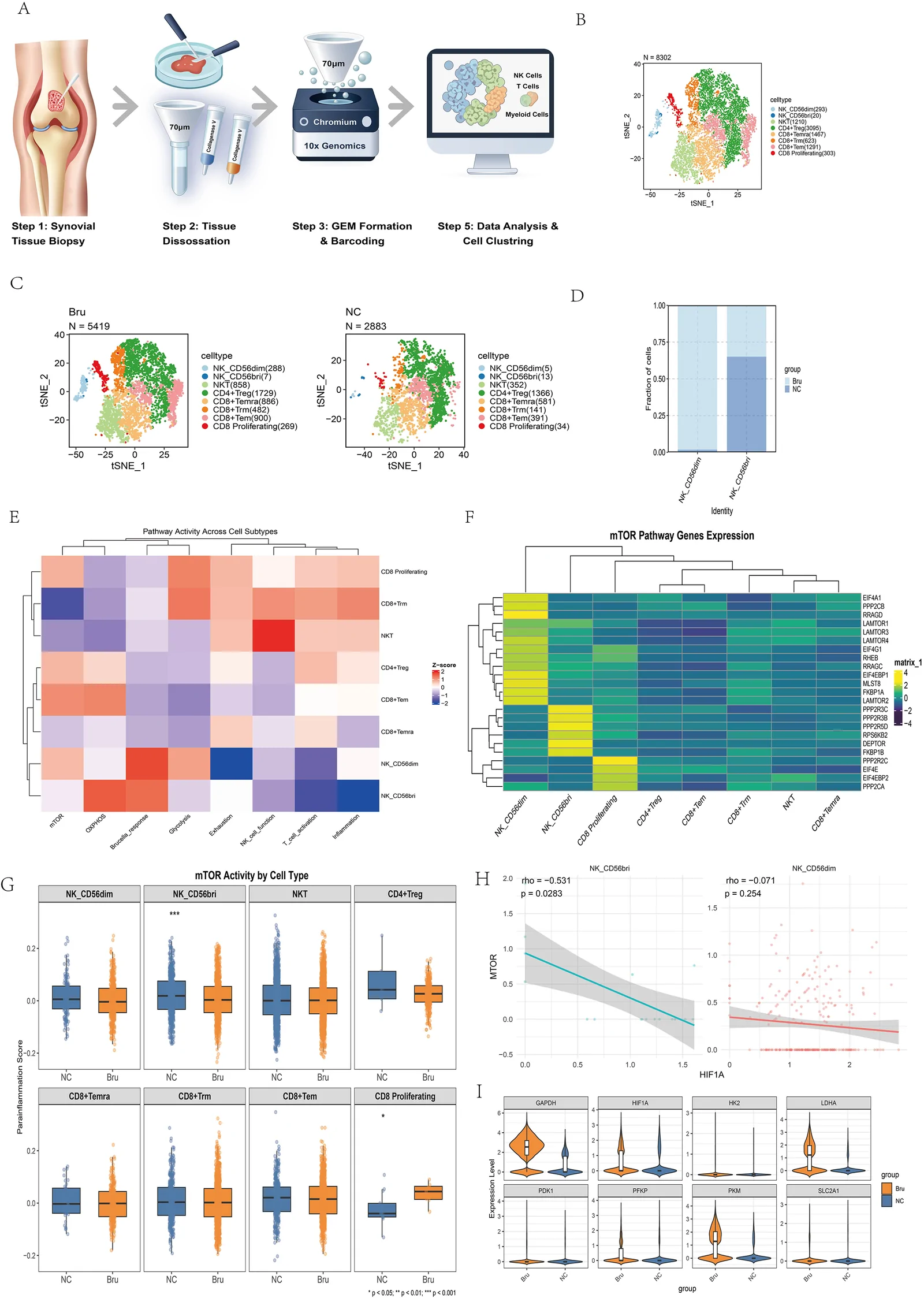
Enrichment and metabolic abnormalities of NK cells in the synovium of brucellar arthritis patients. **(A)** Experimental workflow: from synovial tissue biopsy, tissue dissociation, GEM formation and barcoding, to single-cell data analysis and cell clustering. **(B, C)** t-SNE dimensionality reduction plots showing the distribution of synovial immune cells in healthy controls (Healthy, **(C)**, n=2,883 cells) and chronic brucellar arthritis patients (Bru, C, n=5,419 cells). Cell subpopulations were annotated based on canonical marker expression and are listed with cell numbers: NK_CD56dim (293), NK_CD56bri (20), NKT (1,210), CD4+ regulatory T cells (Treg, 3,095), CD8+ Temra (1,467), CD8+ Trm (623), CD8+ Tem (1,291), and CD8+ proliferating T cells (Proliferating, 303). **(D)** Proportion of NK cells in the synovium of healthy controls versus brucellar arthritis patients. **(E)** Heatmap of pathway activity scores for key signaling pathways across cell subpopulations. Compared with T-cell subsets, NK cell subsets exhibited significantly high activity of the mTOR signaling pathway and oxidative phosphorylation (OXPHOS)-related pathways. **(F, G)** Quantitative comparison of mTOR pathway activity scores among cell subpopulations.**(H)** Distribution of HIF-1α expression levels across cell subpopulations. **(I)** The violin plot displays the expression levels of HIF-1A and glycolysis related genes (SLC2A1, LDHA, PDK1, HK2, PFKP, PKM) in synovial NK cells of healthy controls (NC, n=3) and patients with chronic brucellosis arthritis (Bru, n=4). The data is based on 10x Genomics single-cell transcriptome sequencing (capturing approximately 10000 cells per sample), independent biological replicates of healthy controls (n=3) and chronic phase patients (n=4).Dimensional reduction was performed using UMAP/t-SNE, and pathway activities were calculated by gene set enrichment analysis (GSEA). All data were normalized before visualization.

The t-SNE plots showed the distribution of immune cells in the synovium of healthy controls (Healthy, n=2,883 cells; **Figure 1C**) and chronic brucellar arthritis patients (Bru, n=5,419 cells; **Figure 1C**). Based on canonical marker expression, we identified the following major immune cell subpopulations: NK_CD56dim (293 cells), NK_CD56bri (20 cells), NKT cells (1,210 cells), CD4+ regulatory T cells (Treg, 3,095 cells), CD8+ Temra cells (1,467 cells), CD8+ tissue-resident memory T cells (Trm, 623 cells), CD8+ terminally differentiated T cells (Term, 1,291 cells), and CD8+ proliferating T cells (Proliferating, 303 cells) (**Figure 1C**). The results showed that the proportion of NK cells in the synovium of brucellar arthritis patients was significantly increased compared with healthy controls, suggesting NK cell enrichment in the local inflammatory microenvironment (**Figure 1D**).

To explore the metabolic features of these NK cells, we performed gene set variation analysis (GSVA) to score key signaling pathway activities across cell subpopulations. The pathway activity heatmap (**Figure 1E**) showed that NK cell subsets (especially NK_CD56dim and NK_CD56bri) exhibited significantly higher mTOR signaling and oxidative phosphorylation (OXPHOS) pathway activity scores than T-cell subsets. Quantitative comparisons further confirmed that NK cell subsets had significantly higher mTOR activity scores than other T-cell subsets (**Figures 1F, G**; p < 0.001), and this result was validated by two independent analytical methods. This result suggests that although NK cells are increased in number and exhibit relatively active mTOR signaling in the joint microenvironment of brucellar arthritis, this phenotype may be simultaneously modulated by inhibitory signals.

To further explore potential metabolic inhibitory mechanisms in NK cells, we analyzed the distribution of HIF-1α expression across cell subpopulations and evaluated its correlation with mTOR pathway activity. Spearman correlation analysis revealed a significant negative correlation between HIF-1α expression and mTOR activity in the NK_CD56bri subset (rho = –0.531, p = 0.0283); in the NK_CD56dim subset, the correlation did not reach statistical significance (rho = –0.071, p = 0.254); no significant correlations were observed in other T/NKT subsets (**Figure 1H**). Of note, the NK_CD56bri subset contained very limited cell numbers (only 7–20 cells per group); although the correlation analysis for this subset suggested a potential trend, the statistical robustness is insufficient, and the findings should be considered exploratory.

To clarify the transcriptional differences of HIF1A and key glycolytic genes between healthy controls and chronic-phase patients, we extracted the expression levels of HIF1A, SLC2A1, LDHA, PDK1, HK2, PFKP, and PKM from synovial NK cells of both groups and generated violin plots for comparison. The results (**Figure 1I**) showed no significant differences in mRNA expression for any of these genes between the two groups (*p* > 0.05), indicating that HIF-1α functional activation occurs primarily at the post-translational protein stability level rather than transcriptional upregulation. This finding is highly consistent with the HIF-1α regulatory mechanism mediated by oxygen-dependent prolyl hydroxylase (PHD) degradation, and further confirms that Brucella antigens drive HIF-1α-mediated metabolic suppression primarily through protein stabilization rather than transcriptional reprogramming.

### 3.2 Peripheral blood NK cell functional inhibition in brucellar arthritis correlates with HIF-1α/mTOR pathway suppression

To verify whether the metabolic abnormalities observed in single-cell sequencing were accompanied by functional changes, we further examined the phenotypes and functional characteristics of peripheral blood NK cells from healthy controls (HC), acute-phase brucellar arthritis (A-BA), and chronic-phase brucellar arthritis (C-BA) patients. Flow cytometry results showed that, compared with HC, the proportion of CD3^-^CD56^+^ NK cells in peripheral blood was significantly increased in both acute- and chronic-phase patients (**Figures 2A, B**). However, despite the increase in NK cell numbers, their effector functions were significantly impaired in chronic-phase patients, whereas acute-phase patients exhibited enhanced NK cell activity consistent with immune activation during acute infection (10, 32). Specifically, the percentages of IFN-γ-positive and CD107a-positive NK cells in chronic-phase patients were significantly lower than those in acute-phase patients, whereas acute-phase patients exhibited increased IFN-γ and CD107a positivity compared with HC, consistent with the immune activation characteristics during acute infection; this indicated that NK cells in the chronic phase had entered a functionally suppressed state (**Figures 2A, B**).

**Figure 2:**
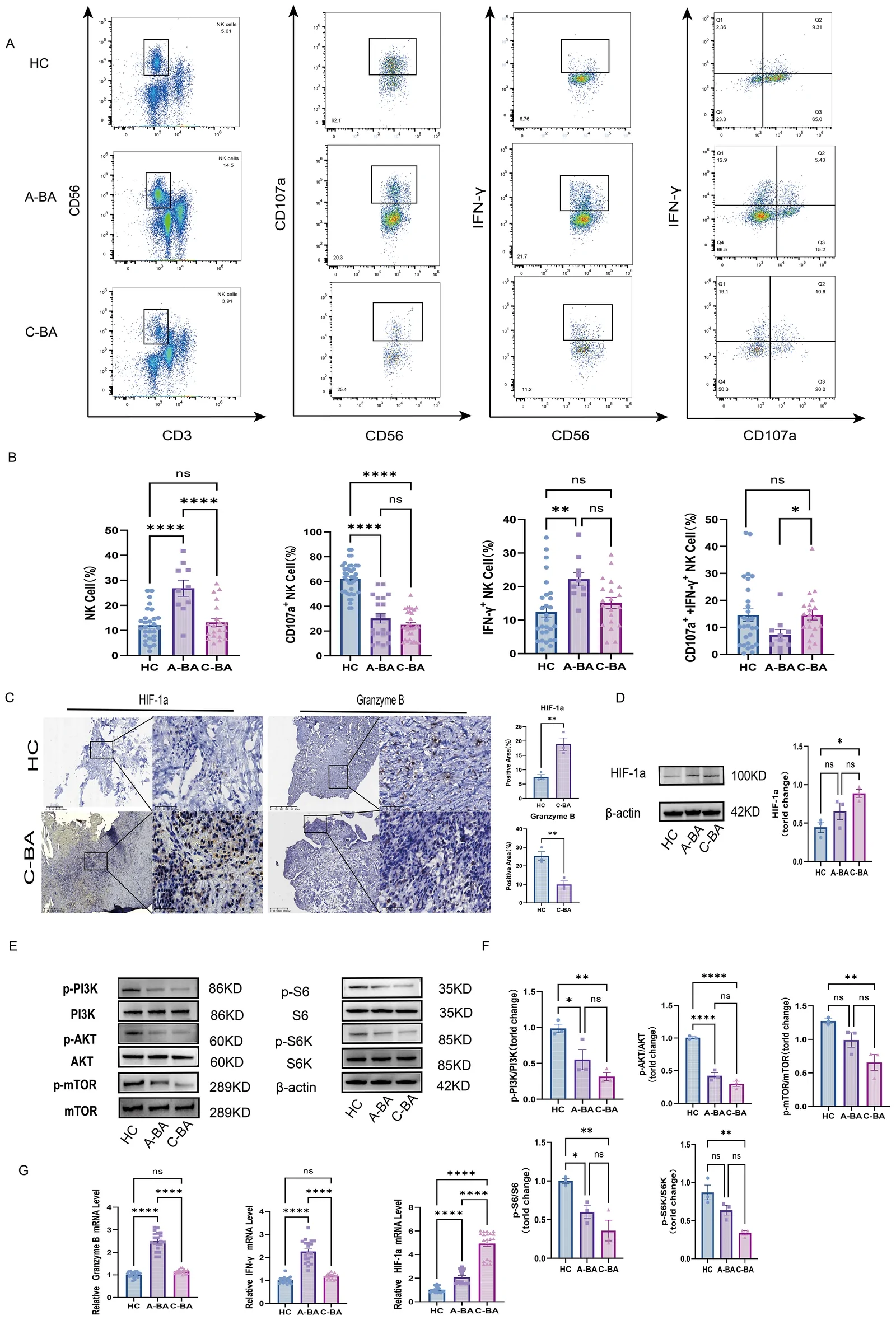
Peripheral blood NK cell functional inhibition in brucellar arthritis correlates with HIF-1α/mTOR pathway suppression. **(A, B)** Flow cytometric analysis of the proportion of CD3^-^CD56^+^ NK cells in peripheral blood from each group (healthy controls, HC; acute-phase, A-BA; chronic-phase, C-BA). Compared with HC, the proportion of NK cells was increased in both acute- and chronic-phase patients. Flow cytometry also detected IFN-γ production and degranulation function (CD107a positivity) of NK cells in each group. NK cells from chronic-phase patients exhibited significantly lower IFN-γ and CD107a expression levels than those from acute-phase patients. **(C)** Expression levels of HIF-1α and Granzyme B in synovial tissues from each group. Chronic-phase patients showed elevated HIF-1α and reduced Granzyme **(B) (D)** Western blot analysis of HIF-1α protein expression in patients from each group. HIF-1α protein bands were markedly enhanced in chronic-phase patients. **(E, F)** Western blot detection of phosphorylation levels of the PI3K/AKT/mTOR pathway (p-PI3K, p-AKT, p-mTOR) and total mTOR in NK cells from each group. Compared with HC, chronic-phase patients had significantly reduced levels of p-PI3K, p-AKT, and p-mTOR. **(G)** Relative mRNA expression levels of Granzyme B, IFN-γ, and HIF-1α in NK cells from each group (qPCR results).Data are presented as mean ± SD (n=3 per group for Western blot/qPCR, n=20 per group for flow cytometry, or at least 3 independent experiments). *P < 0.05, **P < 0.01, ***P < 0.001, ****P < 0.0001; ns, not significant. Statistical methods: one‐way ANOVA with Tukey’s post‐hoc test (after Shapiro‐Wilk normality check) or Student’s t‐test as appropriate; for multiple comparisons, P values were adjusted using the Benjamini‐Hochberg method.

To investigate the molecular mechanism of NK cell function inhibition, we detected the expression levels of HIF-1 α and Granzyme B in the synovial membrane of healthy controls (HC, n=3) and patients with chronic phase Brucella arthritis (C-BA, n=3).The results showed that chronic-phase patients had significantly elevated HIF-1α expression and reduced Granzyme B expression (**Figure 2C**). Western blot analysis further confirmed that HIF-1α protein bands were markedly enhanced in NK cells from chronic-phase patients, whereas HIF-1α expression was relatively low in HC and acute-phase patients (**Figure 2D**). Meanwhile, qPCR results demonstrated that HIF-1α mRNA levels were significantly upregulated in NK cells from chronic-phase patients, while Granzyme B and IFN-γ mRNA levels were significantly downregulated (**Figure 2G**).

It should be noted that although the effector function of peripheral blood NK cells in chronic phase patients is significantly lower than that in acute phase patients (**Figures 2B, G**), their levels are basically equivalent to those in the healthy control group in ex vivo testing without *in vitro* stimulation. This observation indicates that peripheral blood NK cells in chronic phase patients retain similar basal functional capacity as healthy individuals, but show a significant decline relative to the activated state in the acute phase, rather than absolute functional loss below the healthy baseline. Therefore, this article uses the term ‘functional inhibition’ to describe this relative inhibition relative to the acute phase, which may reflect adaptive changes in the immune system to chronic antigen exposure rather than complete functional exhaustion.

Given that HIF-1α can regulate immune cell metabolism through mTOR pathway suppression in the tumor microenvironment (33), we further examined the activation status of the PI3K/AKT/mTOR signaling pathway in NK cells across all groups (34). Western blot results showed that, compared with HC, the phosphorylation levels of p-PI3K, p-AKT, and p-mTOR in NK cells from chronic-phase patients were significantly reduced, while total PI3K, AKT, and mTOR protein expression remained unchanged (**Figures 2E, F**). This result indicated that chronic-phase NK cells exhibited a feature of HIF-1α overexpression accompanied by mTOR pathway phosphorylation inhibition, which was highly consistent with the “negative correlation between HIF-1α and mTOR in NK cells” trend observed in single-cell sequencing.

In summary, peripheral blood NK cells in brucellar arthritis patients displayed a paradoxical phenomenon of “increased numbers with functional inhibition,” which was most prominent in the chronic phase. This functional inhibition was closely associated with sustained HIF-1α protein overexpression and significant suppression of phosphorylation of key mTOR pathway molecules, suggesting that the HIF-1α/mTOR signaling axis may drive NK cells from an immune-activated state in the acute phase toward a functionally suppressed state in the chronic phase by regulating metabolic reprogramming.

To further validate the dynamic changes in NK cell functional status, we employed ELISA to measure the serum concentrations of multiple cytokines in healthy controls (HC), acute-phase (A-BA), and chronic-phase (C-BA) patients, in order to systematically evaluate the systemic immune response characteristics at different stages of brucellar arthritis.

The results showed that, compared with HC, serum levels of IFN-γ and Granzyme B were significantly elevated in acute-phase patients (**Figures 3A, B**), suggesting that NK cells were strongly activated and released large amounts of effector molecules during acute infection to combat Brucella invasion (35, 36). Meanwhile, IL-10, IL-13, and IL-18 were also increased to varying degrees in acute-phase patients, indicating robust immune activation and inflammatory regulatory responses during the acute phase (**Figures 3A, B**). TNF - α showed significant changes among the groups, indicating that Brucella infection may drive TNF - α mediated inflammatory damage through specific mechanisms (**Figures 3A, B**).

**Figure 3:**
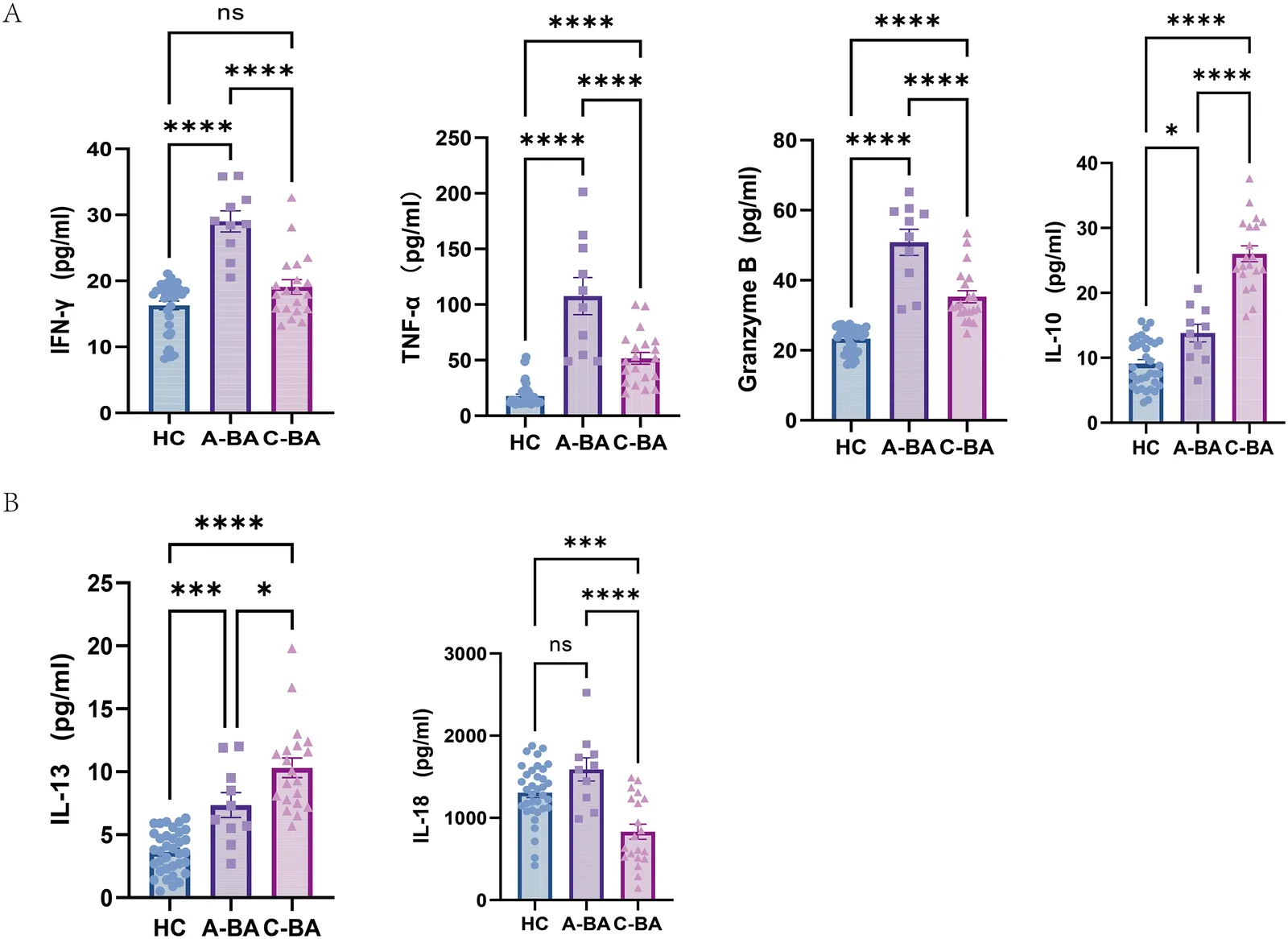
Serum cytokine profiles in healthy controls and brucellar arthritis patients at different disease phases. **(A, B)** Concentrations (pg/mL) of IFN-γ, TNF-α, Granzyme B, IL-10, IL-13, and IL-18 in serum from healthy controls (HC), acute-phase (A-BA), and chronic-phase (C-BA) patients. Data are presented as mean or range (n=20 per group). *P < 0.05, **P < 0.01, ***P < 0.001, ****P < 0.0001; ns, not significant. Statistical methods: one-way ANOVA with Tukey’s post-hoc test (after normality check) or Kruskal-Wallis test as appropriate; for multiple comparisons, P values were adjusted using the Benjamini-Hochberg method.

However, as the disease progressed to the chronic phase, serum IFN-γ and Granzyme B levels significantly declined to near HC levels (**Figures 3A, B**), displaying a typical dynamic evolution pattern of “acute-phase activation, chronic-phase exhaustion” (37). This result was highly consistent with the peripheral blood NK cell functional assay findings, further confirming that NK cell effector functions were severely impaired in the chronic phase. Notably, IL‐10 remained at relatively high levels in chronic‐phase patients, whereas IL‐18 levels were significantly decreased compared with both healthy controls and acute‐phase patients (**Figures 3A, B**). This distinct cytokine profile suggests that while regulatory inflammation persists during the chronic phase, the IL‐18‐associated inflammatory response is not sustained, which may reflect complex immunomodulatory changes associated with Brucella immune evasion and ongoing local joint inflammation.

### 3.3 Heat-killed Brucella melitensis 16M inhibits the mTOR pathway and induces HIF-1α expression in NK cells in a dose- and time-dependent manner

To investigate the effects of whole Brucella components on NK cell function, we treated NK-92 cells *in vitro* with heat-killed Brucella melitensis 16M (HKBA) at various multiplicities of infection (MOI: 0, 1, 10, 50, 100, 200, 500) for 24, 48, or 72 h. First, CCK-8 assays showed that HKBA inhibited NK-92 cell viability in a clear MOI- and time-dependent manner. In the 24 h treatment group, cell viability showed no significant difference from the control group (**Figure 4A**). As the treatment time was extended to 48 h and 72 h, cell viability at the same MOI further decreased. When the treatment time reached 48 h, significant reduction in cell viability began at MOI ≥ 50, and the inhibitory effect gradually intensified with increasing concentrations (**Figure 4A**).

**Figure 4:**
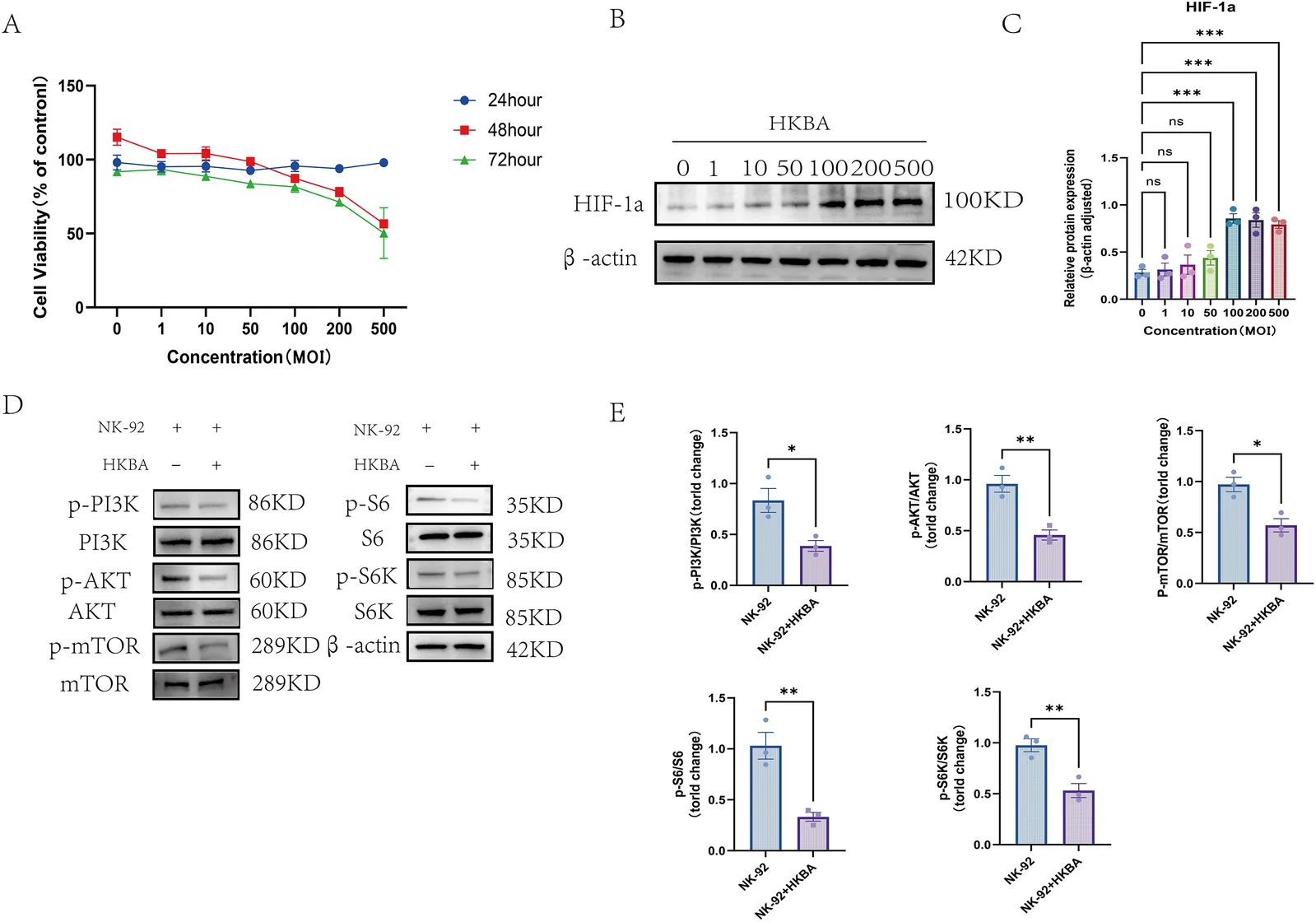
Dose- and time-dependent effects of heat-killed Brucella melitensis 16M (HKBA) on NK-92 cell viability, HIF-1α expression, and mTOR pathway phosphorylation. **(A)** MOI- and time-dependent effects of HKBA on NK-92 cell viability. NK-92 cells were treated with HKBA at different multiplicities of infection (MOI: 0, 1, 10, 50, 100, 200, 500) for 24, 48, or 72 h, and cell viability was assessed by CCK-8 assay. **(B)** MOI-dependent effects of HKBA on HIF-1α protein expression in NK-92 cells. **(C)** Quantitative analysis of HIF-1α protein expression. **(D)** Effects of HKBA on phosphorylation of mTOR signaling pathway proteins in NK-92 cells. **(E)** Quantitative analysis of phosphorylated proteins in the mTOR pathway. Data are presented as fold change, mean ± SD (n=3). Statistical analysis was performed using independent-samples t-test. *P < 0.05, **P < 0.01, ***P < 0.001, ****P < 0.0001; ns, not significant.

We further examined the effect of HKBA on HIF-1α protein expression. Western blot results showed that as the MOI of HKBA increased (0–500), HIF-1α protein expression levels gradually rose (**Figure 4B**). Quantitative analysis indicated that, compared with MOI = 0, HIF-1α expression showed no significant change at MOI = 1 and 10, but began to be significantly upregulated at MOI = 50, and continued to increase at MOI = 100, 200, and 500 (**Figure 4C**). These results suggest that whole Brucella components can induce aberrant HIF-1α overexpression in NK cells, and this effect exhibits a clear dose-dependent manner.

Given that HIF-1α can regulate immune cell metabolism by inhibiting mTOR activity in the tumor microenvironment, we further examined the activation status of the mTOR signaling pathway in NK-92 cells after HKBA treatment. Western blot results showed that, compared with the untreated group (MOI = 0), the HKBA-treated group (MOI = 100) exhibited significantly reduced phosphorylation levels of key molecules in the PI3K/AKT/mTOR pathway, including p-PI3K, p-AKT, and p-mTOR, while total protein expression levels of each molecule remained unchanged (**Figure 4D**). Meanwhile, the phosphorylation levels of the downstream effector molecules p-S6 and p-p70S6K were also correspondingly decreased (**Figure 4D**).

Quantitative analysis further confirmed that the ratios of p-PI3K/PI3K, p-AKT/AKT, p-mTOR/mTOR, p-S6/S6, and p-p70S6K/p70S6K were all significantly lower in the HKBA-treated group than in the untreated group, and this inhibitory effect reached a plateau at MOI = 200 and 500 (**Figure 4E**). This indicates that HKBA not only inhibited upstream kinase activity of the mTOR pathway but also blocked the phosphorylation of its downstream effector molecules, thereby comprehensively suppressing mTOR signaling pathway activation.

In summary, *in vitro* experiments confirmed that heat-killed Brucella melitensis 16M can directly act on NK cells to induce HIF-1α overexpression in an MOI- and time-dependent manner, and comprehensively suppress mTOR signaling pathway activity. This result is highly consistent with the “HIF-1α overexpression with mTOR pathway suppression” phenotype observed in patient samples, suggesting that Brucella infection can directly regulate the HIF-1α/mTOR signaling axis in NK cells through the bacterial body itself (including outer membrane proteins, LPS, and various other components), thereby laying the molecular foundation for subsequent metabolic reprogramming and functional inhibition.

### 3.4 HIF-1α knockout restores NK cell metabolic phenotype and cytotoxic function

To determine the critical role of HIF-1α in Brucella-induced NK cell functional inhibition, we constructed a HIF-1α-knockout (KO) NK-92 cell line using CRISPR/Cas9 technology (**Figure 5**), and systematically compared the effector functions of wild-type (WT) and KO cells under different stimulation conditions by flow cytometry.

**Figure 5:**
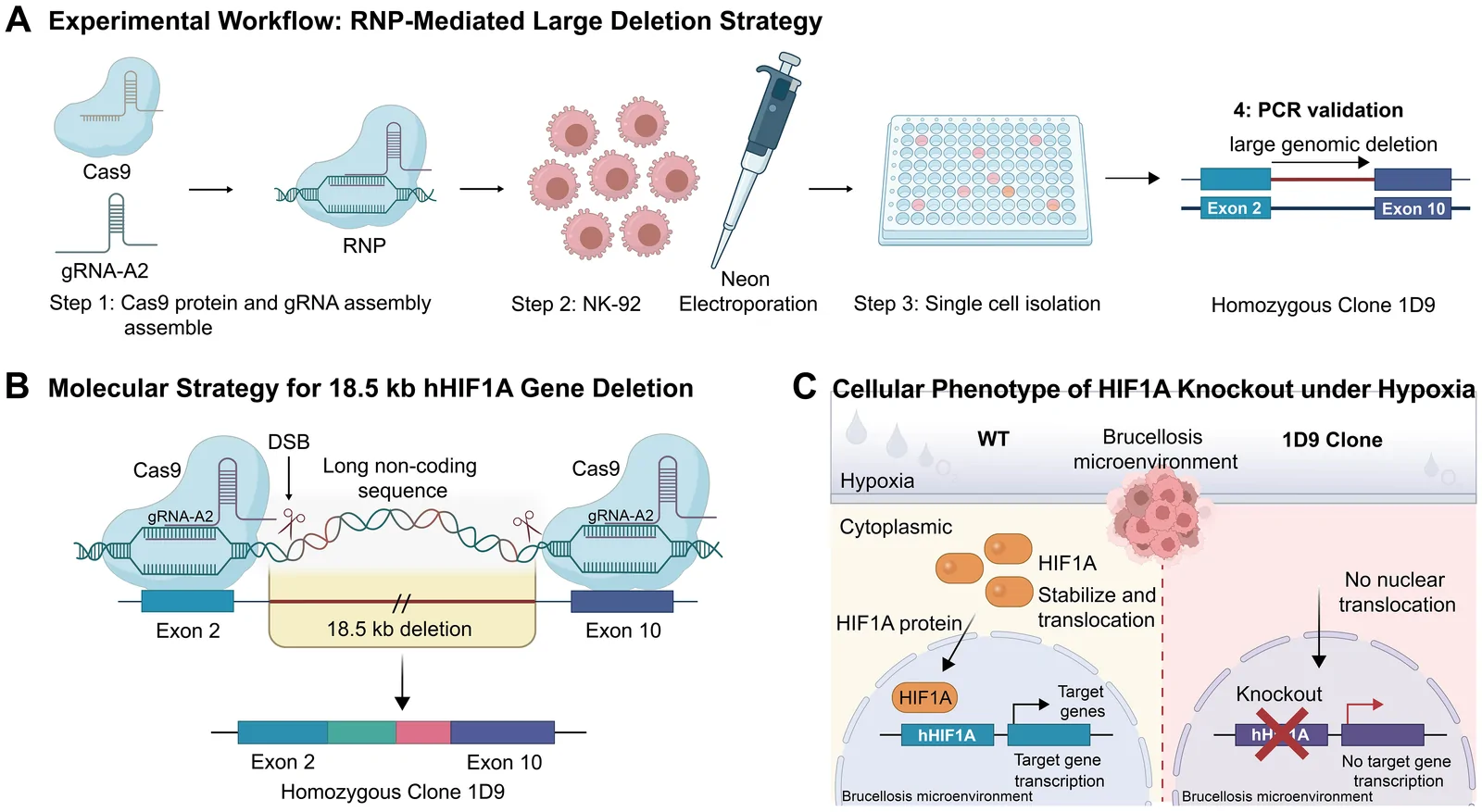
Generation of HIF-1α-knockout NK-92 cells using CRISPR/Cas9. **(A)** Experimental workflow for RNP-mediated large-fragment gene deletion (Experimental Workflow: RNP-Mediated Large Deletion Strategy). Step 1: Cas9 assembly with guide RNAs. Step 2: Electroporation of NK-92 cells. Step 3: Single-cell isolation and clone establishment. Step 4: PCR genotyping. **(B)** Molecular strategy for 18.5 kb deletion of the human HIF1A gene (Molecular Strategy for 18.5 kb hHIF1A Gene Deletion). gRNA-A1 targets downstream of exon 2, and gRNA-A2 targets upstream of exon 10. The two RNP complexes respectively induce double-strand breaks (DSBs) in the genomic DNA; direct joining with homozygous deletion of the intervening 18.5 kb sequence yields knockout clone 1D9, which disrupts the complete coding sequence of HIF1A at the genomic level. **(C)** Cellular phenotype of HIF-1α knockout under hypoxia (Cellular Phenotype of HIF1A Knockout under Hypoxia). Left: Wild-type (WT) cells—under hypoxia (low oxygen tension) in the Brucellosis microenvironment, HIF-1α protein stably accumulates in the cytoplasm, translocates to the nucleus, binds to target gene promoters, and initiates transcription of downstream hypoxia-responsive target genes. Right: 1D9 knockout clone—the HIF1A gene is knocked out (red crosses indicate gene inactivation), no functional HIF-1α protein is synthesized, no HIF-1α nuclear translocation occurs, and downstream hypoxia-target genes cannot be transcribed, resulting in loss of hypoxia-induced HIF-1 pathway activation.

Flow cytometry was used to detect the percentage of IFN-γ-positive cells in each group of NK cells. WT and HIF-1α KO NK-92 cells were respectively co-cultured with K562 target cells (K562+NK-WT, K562+NK-KO), or cultured after HKBA treatment (MOI = 100) (HKBA+NK-WT, HKBA+NK-KO), and intracellular IFN-γ expression was examined. The results showed that under co-culture with K562 target cells, the IFN-γ positivity rate of WT NK-92 cells was approximately 4–5%, whereas that of HIF-1α KO cells was significantly elevated to approximately 32–33% (**Figure 6A**). HKBA treatment significantly suppressed IFN-γ production in WT cells, with the positivity rate decreasing to approximately 12–13%; however, HIF-1α KO cells maintained a relatively high IFN-γ positivity rate of approximately 41–42% after HKBA treatment (**Figure 6A**). It should be noted that under HKBA stimulation conditions, the positive rates of IFN - γ and CD107a in HIF-1 α KO cells were significantly higher than those in WT cells (**Figures 6A, B**), indicating that HIF-1 α deficiency can effectively counteract the functional limitation caused by Brucella antigen exposure. It should be noted that the level of CD107a in HKBA+NK-WT cells is not lower than that in K562+NK-WT cells; These two groups represent completely different experimental conditions - K562+NK-WT reflects the direct killing ability of target cells under antigen free pre exposure, while HKBA+NK-WT reflects the basic functional state of HKBA after long-term stimulation (38). The core finding of this section is that KO cells consistently outperformed WT cells under all conditions, with the HKBA treatment group showing the most significant difference - KO cells maintained high functional levels under HKBA exposure (76-78% vs. 35-36%, P<0.001). This indicates that HIF-1 α knockout confers resistance to HKBA induced functional impairment in NK cells, rather than simply enhancing basal activity (39).

**Figure 6:**
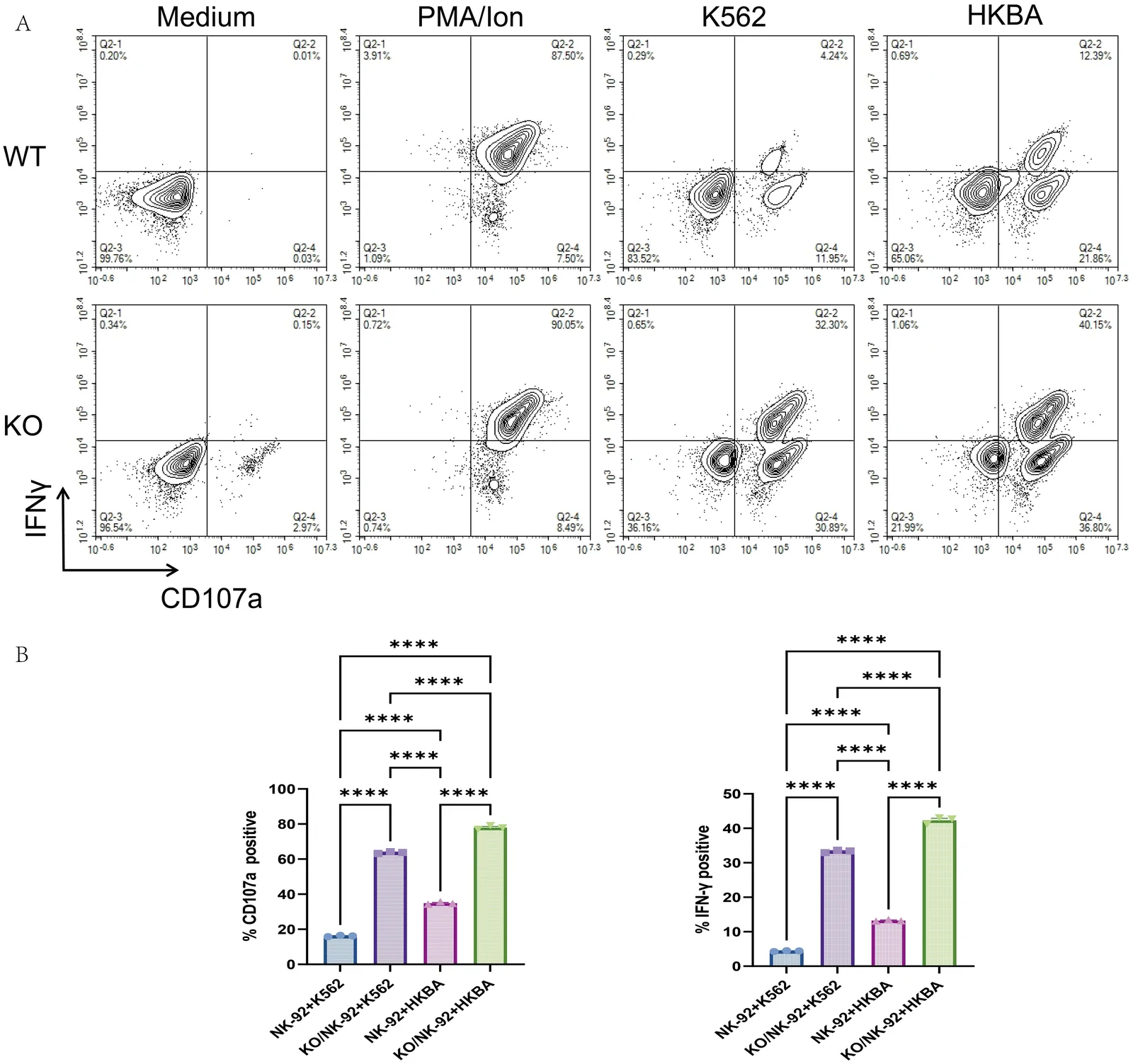
HIF-1α knockout restores NK cell effector functions and counteracts HKBA-induced suppression. **(A)** Flow cytometric analysis of the percentage of IFN-γ-positive cells in each group of NK cells. NK-92 cells or HIF-1α-knockout (KO) NK-92 cells were co-cultured with K562 target cells, or cultured after HKBA treatment (MOI = 100), and intracellular IFN-γ and CD107a expression were detected. **(B)** Detection results of NK cell degranulation function (CD107a positivity rate) in each group. Data are presented as mean ± SD (n=3 independent experiments). Statistical analysis was performed using two-way ANOVA or t-test. *P < 0.05, **P < 0.01, ***P < 0.001, ****P < 0.0001; ns, not significant.

We further examined the degranulation function (CD107a positivity percentage) of NK cells in each group. Grouping and treatments were the same as in (A). Consistent with the IFN-γ results, under co-culture with K562 target cells, the CD107a positivity rate of WT NK-92 cells was approximately 16–17%, whereas that of HIF-1α KO cells was significantly elevated to approximately 62–63% (**Figure 6B**). After HKBA treatment, the CD107a positivity rate of WT cells decreased to approximately 35–36%, while HIF-1α KO cells maintained a relatively high CD107a positivity rate of approximately 76–78% after HKBA treatment (**Figure 6B**). This indicated that HIF-1α knockout significantly enhanced NK cell degranulation capacity and effectively antagonized HKBA-induced functional suppression.

In summary, HIF-1α knockout significantly restored NK cell cytotoxic function, manifesting as comprehensive enhancement of IFN-γ secretion and CD107a degranulation capacity. More importantly, under conditions of HKBA-simulated Brucella infection (40), HIF-1α KO cells maintained relatively high levels of effector function, suggesting that Brucella-induced HIF-1α overexpression is a key factor leading to NK cell metabolic and functional inhibition. This finding laid an important foundation for subsequent exploration of the molecular mechanisms by which the HIF-1α/mTOR axis regulates NK cell metabolic reprogramming.

#### 3.4.1 Transcriptomic profiling of NK cells after HIF-1α knockout

To comprehensively dissect the downstream molecular network through which HIF-1α regulates NK cell metabolism and function, we performed RNA-seq analysis on the transcriptomes of wild-type (WT) and HIF-1α-knockout (KO) NK-92 cells after treatment with HKBA (MOI = 100) for 72 h (three biological replicates per group). Differential expression analysis revealed that, compared with the WT group, the KO group had a total of 467 significantly upregulated genes and 514 significantly downregulated genes. The volcano plot visually presented the distribution characteristics of upregulated and downregulated genes (**Figure 7A**), indicating that HIF-1α deletion profoundly remodeled the transcriptomic landscape of NK cells.

**Figure 7:**
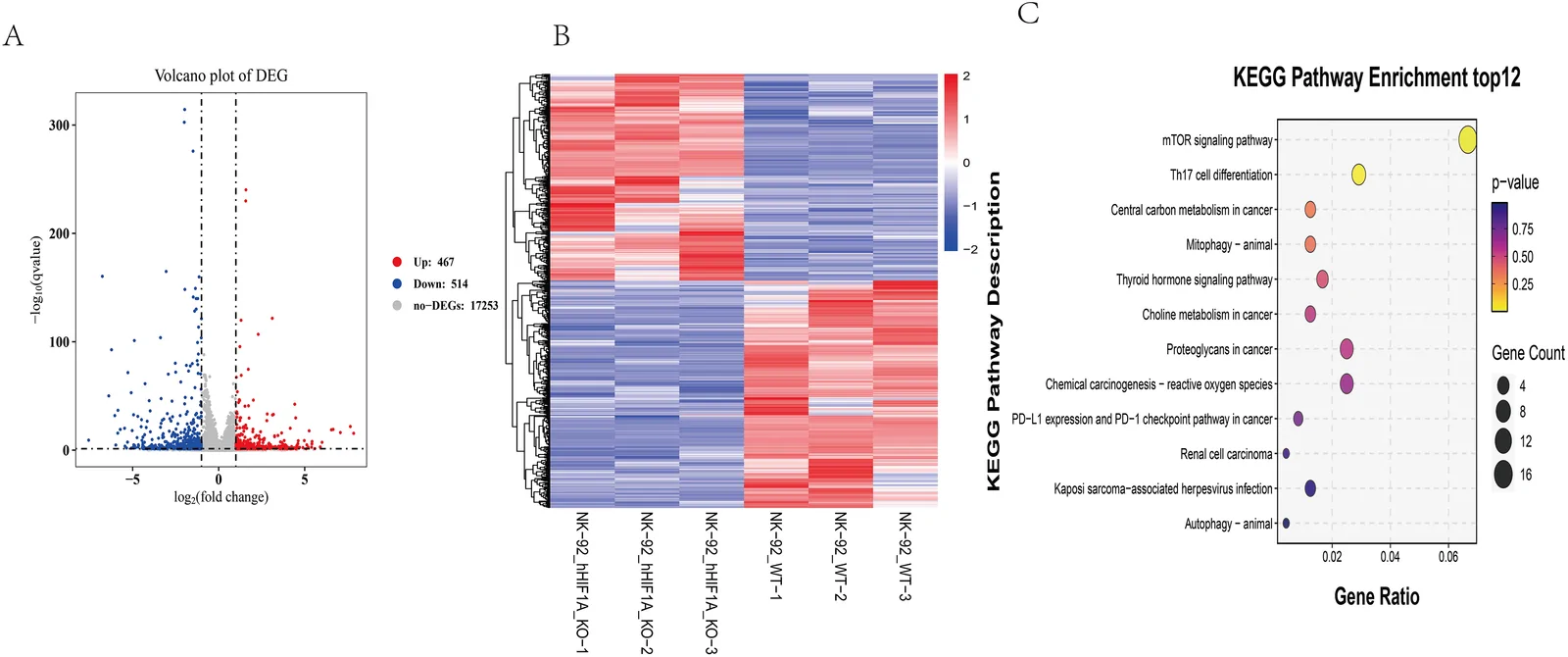
Transcriptomic profiling of WT and HIF-1α-KO NK-92 cells after HKBA treatment. **(A)** Volcano plot showing differentially expressed genes between wild-type (WT) and HIF-1α-knockout (KO) NK-92 cells treated with HKBA (MOI = 100) for 72 (h) **(B)** Heatmap showing clustering of WT and KO samples at the transcriptomic level. **(C)** KEGG pathway enrichment analysis results, showing that upregulated genes in the KO group were significantly enriched in multiple energy metabolism and signaling pathways. Data are based on three independent biological replicates.

To understand the expression patterns of these differentially expressed genes across samples, we performed hierarchical clustering analysis on the significantly differentially expressed genes (**Figure 7B**). The heatmap results showed that WT and KO samples formed two distinct major clusters at the transcriptomic level, with three biological replicates within each group tightly clustered, indicating good consistency and reproducibility of the sequencing data.

KEGG pathway enrichment analysis showed that upregulated genes in the KO group were significantly enriched in multiple metabolic and signaling pathways (**Figure 7C**). Among them, the enrichment of mTOR signaling pathway is the most significant, indicating that the activity of mTOR pathway is deeply activated at the transcriptional level after HIF-1 α knockout.

In summary, the transcriptomic sequencing results confirmed at the genome-wide level that HIF-1α knockout, through deep activation of the mTOR signaling pathway (41), reversed the Brucella-induced metabolic inhibition and functional suppression phenotype of NK cells, providing direct transcriptomic evidence for the subsequent regulatory mechanism of the HIF-1α/mTOR axis.

### 3.5 HIF-1α mediates NK cell functional decline through mTOR pathway inhibition

To further validate the central role of the HIF-1α/mTOR signaling axis in regulating NK cell metabolic reprogramming (42), we treated wild-type (WT) NK-92 cells and HIF-1α-knockout (KO) NK-92 cells with HKBA (MOI = 100) for 72 h, or left them untreated as controls, and systematically compared mTOR pathway activity, mitochondrial respiratory function, glycolytic capacity, and effector molecule expression across groups (43).

Western blot analysis was performed to detect the phosphorylation levels of the PI3K/AKT/mTOR signaling pathway and downstream effector molecules in each group (**Figure 8A**). The results showed that HKBA treatment significantly suppressed the phosphorylation levels of p-PI3K, p-AKT, p-mTOR, and the downstream effector molecules p-S6 and p-S6K in WT NK-92 cells, while total protein expression remained unchanged. However, in HIF-1α KO cells, the inhibitory effect of HKBA treatment on mTOR pathway phosphorylation was significantly attenuated, with each phosphorylated protein showing marked recovery.

**Figure 8:**
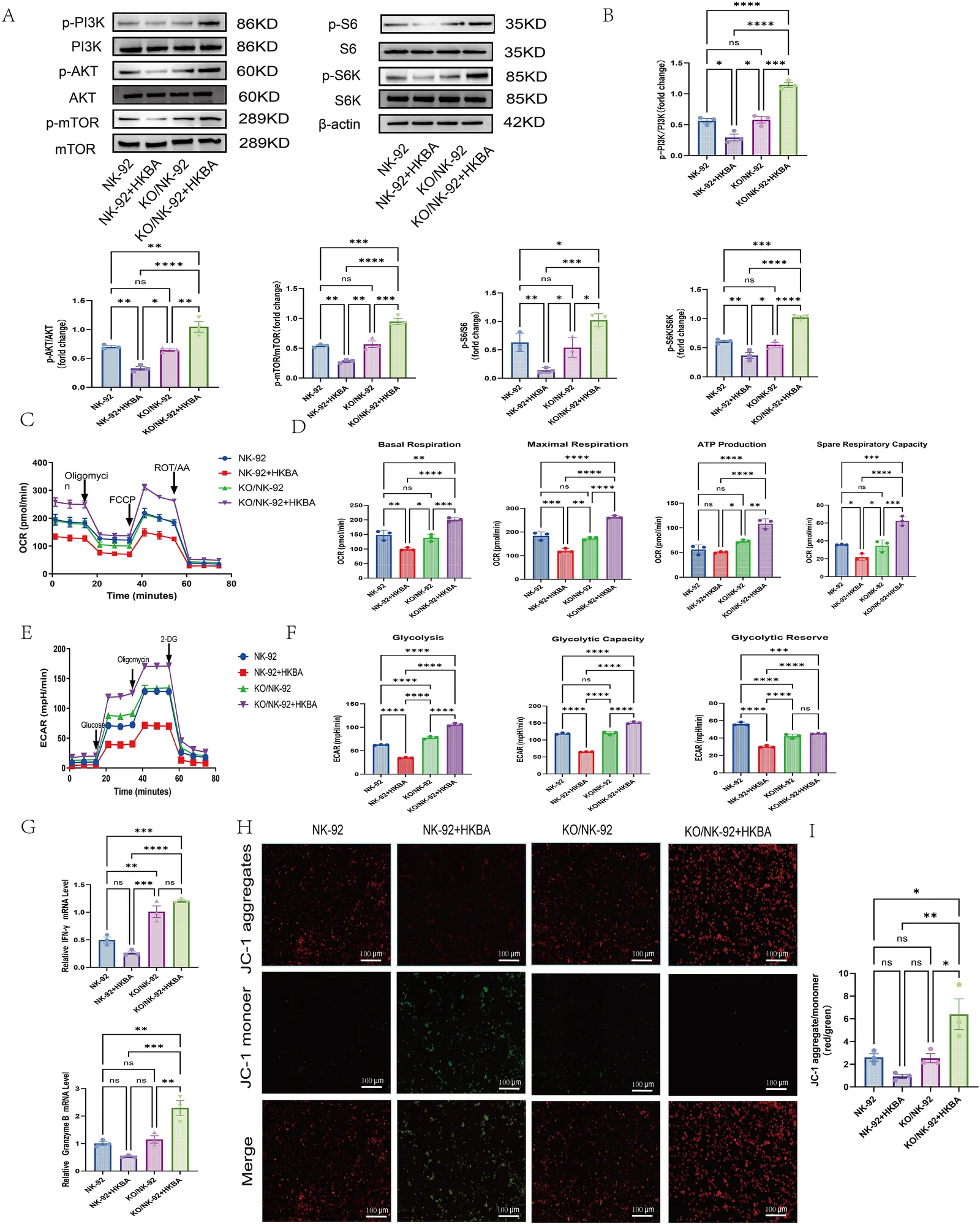
HIF‐1α knockout restores mTOR pathway activity, mitochondrial respiration, glycolysis, and effector molecule expression in NK‐92 cells after HKBA treatment. Wild‐type (WT) NK‐92 cells and HIF‐1α‐knockout (KO) NK‐92 cells were treated with HKBA (MOI = 100) for 72 h, or left untreated as controls. **(A)** Western blot analysis of phosphorylation levels of the PI3K/AKT/mTOR signaling pathway and downstream effector molecules in each group. **(B)** Quantitative analysis of the ratios of phosphorylated proteins to total proteins (fold change).**(C)** Oxygen consumption rate (OCR) measured by Seahorse XFe96 metabolic analyzer, reflecting mitochondrial oxidative phosphorylation function. OCR was measured in real time after sequential injection of oligomycin (1 μmol/L), FCCP (1 μmol/L), and rotenone/antimycin A (ROT/AA, 0.5 μmol/L). **(D)** Quantitative analysis of OCR parameters based on **(C)**. Data are presented as OCR (pmol/min), mean ± SD (n=3). **(E)** Extracellular acidification rate (ECAR) measured by Seahorse XFe96, reflecting glycolytic activity. ECAR was measured in real time after sequential injection of glucose (10 mmol/L),oligomycin(1μmol/L),and2‐deoxy‐D‐glucose(2‐DG, 50 mmol/L). **(F)** Quantitative analysis of ECAR parameters based on **(E)**. Data are presented as ECAR (mpH/min), mean ± SD (n=3). **(G)** Detection of IFN‐γ and Granzyme B secretion capacity by PCR. **(H)** Representative JC‐1 fluorescence images of NK cells in each group, showing JC‐1 red aggregate channel, green monomer channel, and merged fluorescence images. **(I)** Quantitative analysis of the JC‐1 red/green fluorescence ratio, reflecting mitochondrial membrane potential levels in each group. Data are presented as mean ± SD, and were repeated in three independent experiments. All comparisons were performed using one‐way ANOVA. *P < 0.05, **P < 0.01, ***P < 0.001, ****P < 0.0001; ns, not significant (P > 0.05).

Quantitative analysis further confirmed (**Figure 8B**) that, compared with WT NK-92 cells, the ratios of p-PI3K/PI3K, p-AKT/AKT, p-mTOR/mTOR, p-S6/S6, and p-S6K/S6K in HIF-1α KO cells after HKBA treatment were all significantly elevated. This result indicated that HIF-1α knockout significantly restored the activation status of the mTOR signaling pathway in NK cells and antagonized HKBA-induced pathway suppression.

The Seahorse XFe96 metabolic analyzer was used to measure the oxygen consumption rate (OCR) of cells in each group, reflecting mitochondrial oxidative phosphorylation function. Oligomycin (1 μmol/L), FCCP (1 μmol/L), and rotenone/antimycin A (ROT/AA, 0.5 μmol/L) were sequentially injected, and OCR was measured in real time (**Figure 8C**). The results showed that HKBA treatment significantly reduced basal respiration, maximal respiration, ATP production, and spare respiratory capacity (SRC) in WT NK-92 cells. However, HIF-1α KO cells maintained relatively high OCR levels after HKBA treatment, with their mitochondrial function being significantly protected.

Quantitative analysis based on OCR parameters (**Figure 8D**) showed that, compared with the WT NK-92+HKBA group, the KO/NK-92+HKBA group exhibited significantly higher basal respiration, maximal respiration, ATP production, and spare respiratory capacity, with some parameters recovering to levels close to those of untreated WT cells. This indicated that HIF-1α knockout, through restoration of mTOR pathway activity, significantly improved mitochondrial oxidative phosphorylation function in NK cells.

Furthermore, the Seahorse XFe96 was used to measure the extracellular acidification rate (ECAR) of cells in each group, reflecting glycolytic activity. Glucose (10 mmol/L), oligomycin (1 μmol/L), and 2-deoxy-D-glucose (2-DG, 50 mmol/L) were sequentially injected, and ECAR was measured in real time (**Figure 8E**). The results showed that HKBA treatment significantly suppressed basal glycolysis and glycolytic capacity in WT NK-92 cells. In contrast, HIF-1α KO cells maintained relatively high ECAR levels after HKBA treatment, with glycolytic function being significantly restored.

Quantitative analysis based on ECAR parameters (**Figure 8F**) showed that, compared with the WT NK-92+HKBA group, the KO/NK-92+HKBA group exhibited significantly higher glycolysis, glycolytic capacity, and glycolytic reserve. This result indicated that HIF-1α knockout not only restored mitochondrial oxidative phosphorylation but also simultaneously enhanced NK cell glycolytic capacity, achieving comprehensive reactivation of energy metabolism.

Notably, under baseline conditions without HKBA stimulation, HIF-1α knockout (KO) and wild-type (WT) NK-92 cells showed no significant differences in either OCR or ECAR (**Figures 8C, E**). This observation indicates that HIF-1α is not a constitutive regulator of basal NK cell energy metabolism under resting conditions. Instead, its regulatory role becomes critically engaged specifically upon pathogenic stress (HKBA stimulation), where WT cells exhibit significant metabolic suppression while KO cells maintain robust mitochondrial respiration and glycolysis. This finding further supports our central conclusion that the HIF-1α/mTOR axis is specifically activated during Brucella antigen-induced metabolic reprogramming, rather than reflecting a general, non-specific metabolic defect in the KO cells.

The ability of cells to secrete IFN-γ and Granzyme B was examined by PCR (**Figure 8G**). The results showed that HKBA treatment significantly downregulated the mRNA levels of IFN-γ and Granzyme B in WT NK-92 cells. However, in HIF-1α KO cells after HKBA treatment, the mRNA expression of IFN-γ and Granzyme B was significantly restored, which was highly consistent with the functional assay results.

JC-1 fluorescence imaging and quantitative analysis further verified the mitochondrial damage phenotype (**Figures 8H, I**). After HKBA treatment, WT NK-92 cells exhibited significantly enhanced green fluorescence (JC-1 monomers) and markedly reduced red fluorescence (JC-1 aggregates), with a significant decrease in the red/green fluorescence ratio, suggesting that HKBA disrupted mitochondrial membrane integrity in NK cells. In contrast, HIF-1α KO cells subjected to the same HKBA stimulation showed a significant recovery of red fluorescence intensity, attenuation of green fluorescence signal, and a significantly increased red/green ratio compared with the WT+HKBA group, indicating that HIF-1α knockout significantly reversed the mitochondrial membrane potential collapse induced by Brucella virulence factors and protected mitochondrial homeostasis. This JC-1 quantitative result was completely consistent with the Seahorse mitochondrial respiration OCR parameter changes, providing fluorescence morphological evidence that HIF-1α deletion can restore intact mitochondrial function in NK cells.

To further validate the central role of the HIF-1α/mTOR signaling axis in regulating NK cell metabolic reprogramming, we treated wild-type (WT) NK-92 cells and HIF-1α-knockout (KO) NK-92 cells with HKBA (MOI = 100) for 72 h, or left them untreated as controls. In some experimental groups, cells were pretreated with the mTOR pathway inhibitor rapamycin (100 nM) for 1 h before HKBA addition. We systematically compared mTOR pathway activity, mitochondrial respiratory function, glycolytic capacity, and effector molecule expression across all groups.

Western blot analysis was performed to detect the phosphorylation levels of the PI3K/AKT/mTOR signaling pathway and downstream effector molecules in each group (**Figure 9A**). The results showed that HKBA treatment significantly suppressed the phosphorylation levels of p-PI3K, p-AKT, p-mTOR, and the downstream effector molecules p-S6 and p-S6K in WT NK-92 cells, while total protein expression remained unchanged. However, in HIF-1α KO cells, the inhibitory effect of HKBA treatment on mTOR pathway phosphorylation was significantly attenuated, with each phosphorylated protein showing marked recovery. Importantly, when pretreated with rapamycin, the restored mTOR pathway phosphorylation levels in HIF-1α KO cells were completely blocked, and each phosphorylated protein level fell back to the suppression level observed in the HKBA-alone treatment group.

**Figure 9:**
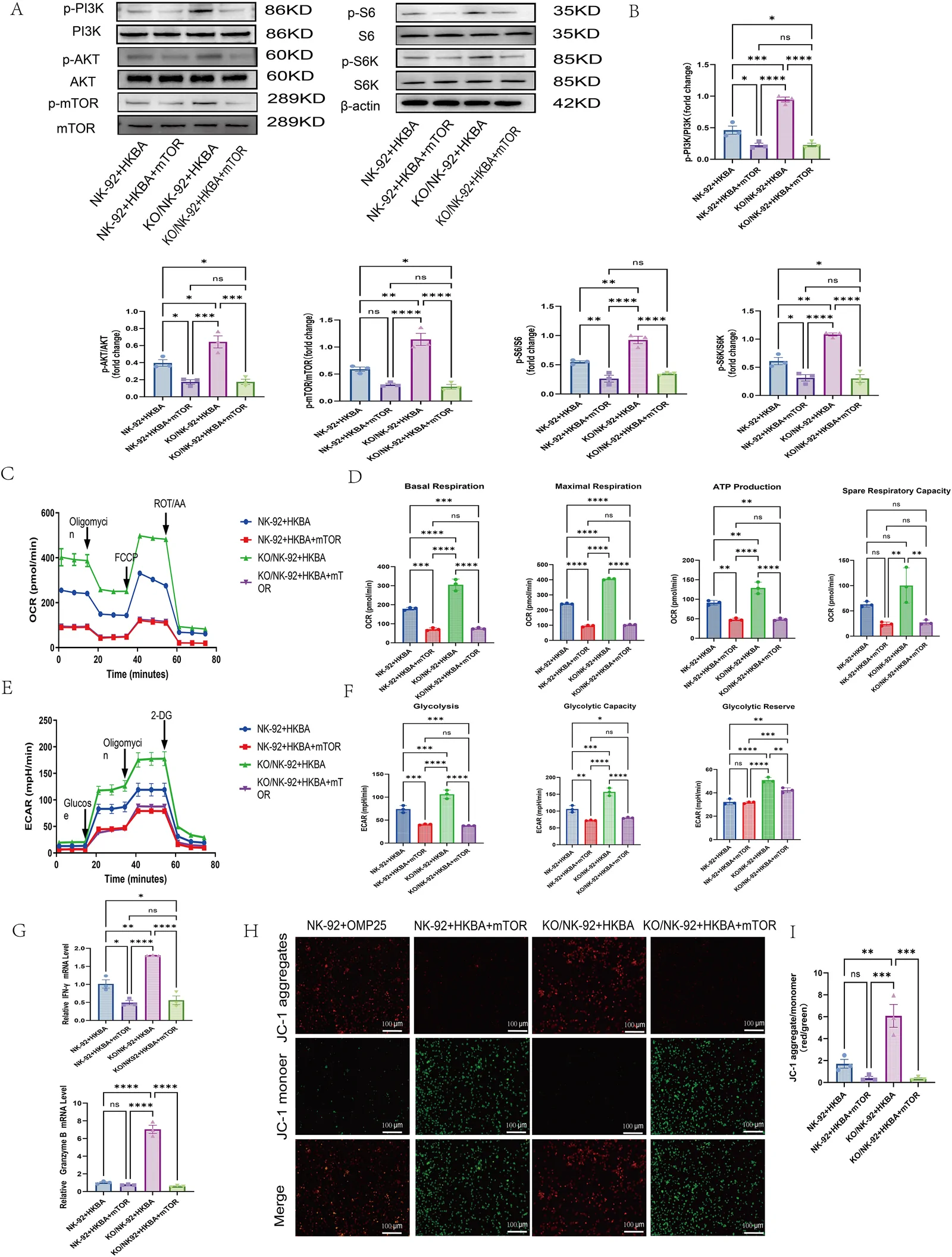
mTOR inhibitor rapamycin completely blocks the restorative effects of HIF-1α knockout on NK cell metabolism and function. Wild-type (WT) NK-92 cells and HIF-1α-knockout (KO) NK-92 cells were treated with HKBA (MOI = 100) for 72 h, or left untreated as controls. In some experimental groups, cells were pretreated with the mTOR pathway inhibitor rapamycin (100 nM) for 1 h before HKBA addition. **(A)** Western blot analysis of phosphorylation levels of the PI3K/AKT/mTOR signaling pathway and downstream effector molecules in each group. **(B)** Quantitative analysis of the ratios of phosphorylated proteins to total proteins (fold change). **(C)** Oxygen consumption rate (OCR) measured by Seahorse XFe96 metabolic analyzer, reflecting mitochondrial oxidative phosphorylation function. OCR was measured in real time after sequential injection of oligomycin (1 μmol/L), FCCP (1 μmol/L), and rotenone/antimycin A (ROT/AA, 0.5 μmol/L). **(D)** Quantitative analysis of OCR parameters based on **(C)**. Data are presented as OCR (pmol/min), mean ± SD (n=3). **(E)** Extracellular acidification rate (ECAR) measured by Seahorse XFe96, reflecting glycolytic activity. ECAR was measured in real time after sequential injection of glucose (10 mmol/L), oligomycin (1 μmol/L), and 2-deoxy-D-glucose (2-DG, 50 mmol/L). **(F)** Quantitative analysis of ECAR parameters based on **(E)**. Data are presented as ECAR (mpH/min), mean ± SD (n=3). **(G)** Detection of IFN-γ and Granzyme B secretion capacity by PCR.**(H)** Representative JC-1 fluorescence images of NK cells in each group, showing JC-1 red aggregate channel, green monomer channel, and merged fluorescence images. **(I)** Quantitative analysis of the JC-1 red/green fluorescence ratio, reflecting mitochondrial membrane potential levels in each group. Data are presented as mean ± SD, and were repeated in three independent experiments. All comparisons were performed using one-way ANOVA. *P < 0.05, **P < 0.01, ***P < 0.001, ****P < 0.0001; ns, not significant (P > 0.05).

Quantitative analysis further confirmed (**Figure 9B**) that, compared with the WT NK-92+HKBA group, the ratios of p-PI3K/PI3K, p-AKT/AKT, p-mTOR/mTOR, p-S6/S6, and p-S6K/S6K in the KO/NK-92+HKBA group were all significantly elevated; however, in the KO/NK-92+HKBA+Rapa group, all these ratios were significantly reduced compared with the KO/NK-92+HKBA group, and showed no significant difference from the NK-92+HKBA group, indicating that rapamycin completely blocked the mTOR pathway restoration mediated by HIF-1α knockout.

The Seahorse XFe96 metabolic analyzer was used to measure the oxygen consumption rate (OCR) of cells in each group, reflecting mitochondrial oxidative phosphorylation function. Oligomycin (1 μmol/L), FCCP (1 μmol/L), and rotenone/antimycin A (ROT/AA, 0.5 μmol/L) were sequentially injected, and OCR was measured in real time (**Figure 9C**). The results showed that HKBA treatment significantly reduced basal respiration, maximal respiration, ATP production, and spare respiratory capacity (SRC) in WT NK-92 cells. HIF-1α KO cells maintained relatively high OCR levels after HKBA treatment, with their mitochondrial function being significantly protected. However, when pretreated with rapamycin, the restored mitochondrial respiratory function in HIF-1α KO cells was completely eliminated, and their OCR curve largely overlapped with that of the NK-92+HKBA+mTOR group.

Quantitative analysis based on OCR parameters (**Figure 9D**) showed that, compared with the NK-92+HKBA group, the KO/NK-92+HKBA group exhibited significantly higher basal respiration, ATP production, maximal respiration, and spare respiratory capacity; however, after rapamycin addition (KO/NK-92+HKBA+mTOR group), all these parameters were significantly lower than those in the KO/NK-92+HKBA group, and showed no significant difference from the NK-92+HKBA group.

Furthermore, the Seahorse XFe96 was used to measure the extracellular acidification rate (ECAR) of cells in each group, reflecting glycolytic activity. Glucose (10 mmol/L), oligomycin (1 μmol/L), and 2-deoxy-D-glucose (2-DG, 50 mmol/L) were sequentially injected, and ECAR was measured in real time (**Figure 9E**). The results showed that HKBA treatment significantly suppressed basal glycolysis and glycolytic capacity in WT NK-92 cells. HIF-1α KO cells maintained relatively high ECAR levels after HKBA treatment, with glycolytic function being significantly restored. However, when pretreated with rapamycin, the restored glycolytic function in HIF-1α KO cells was completely eliminated.

Quantitative analysis based on ECAR parameters (**Figure 9F**) showed that, compared with the NK-92+HKBA group, the KO/NK-92+HKBA group exhibited significantly higher glycolysis, glycolytic capacity, and glycolytic reserve; however, after rapamycin addition (KO/NK-92+HKBA+mTOR group), all these parameters were significantly lower than those in the KO/NK-92+HKBA group, and showed no significant difference from the NK-92+HKBA group.

The ability of cells to secrete IFN-γ and Granzyme B was examined by PCR (**Figure 9G**). The results showed that HKBA treatment significantly downregulated the mRNA levels of IFN-γ and Granzyme B in WT NK-92 cells. In HIF-1α KO cells after HKBA treatment, the mRNA expression of IFN-γ and Granzyme B was significantly restored. However, after rapamycin addition (KO/NK-92+HKBA+mTOR group), the mRNA levels of IFN-γ and Granzyme B were significantly lower than those in the KO/NK-92+HKBA group, and showed no significant difference from the NK-92+HKBA group.

JC-1 fluorescence imaging quantification further confirmed that the mTOR inhibitor completely counteracted the protective effect of HIF-1α knockout on mitochondria (**Figures 9H, I**). The significantly elevated JC-1 red/green fluorescence ratio in the KO+HKBA group dropped sharply after combined rapamycin treatment, with green fluorescence signal markedly increased again and red fluorescence attenuated; the red/green ratio in the KO+HKBA+mTOR group showed no statistical difference from the WT+HKBA group. Fluorescence morphology demonstrated that after mTOR pathway blockade, even in the absence of HIF-1α, NK cell mitochondrial membrane potential still underwent irreversible collapse. This result directly demonstrated that HIF-1α regulation of mitochondrial homeostasis is completely dependent on downstream mTOR pathway activation, and that mTOR signaling inactivation completely abolishes the mitochondrial functional restoration conferred by HIF-1α knockout.

Based on the above in vitro and in vivo experimental results, we propose a working model in which Brucella drives NK cell metabolic reprogramming and functional exhaustion through the HKBA-HIF-1 α - mTOR axis (**Figure 10**). In this model, HKBA directly acts on NK cells, inducing stable accumulation and incorporation of HIF-1 α protein into the nucleus, while significantly inhibiting phosphorylation activation of the PI3K/AKT/mTOR signaling pathway. The sustained decrease in mTOR activity leads to dysfunction of mitochondrial oxidative phosphorylation (OXPHOS) and impaired glycolytic capacity (ECAR), causing NK cells to enter a state of "metabolic rigidity"; Ultimately, the synthesis and release of effector molecules IFN - γ and Granzyme B decreased, the degranulation ability mediated by CD107a decreased, and the anti infective cytotoxicity of NK cells significantly weakened. Knocking out HIF-1 α through CRISPR/Cas9 can release the inhibition of mTOR, restore energy metabolism, and rescue killing function, but this rescue effect can be completely blocked by mTOR inhibitor rapamycin, proving that HIF-1 α mainly achieves the above regulation by inhibiting the mTOR pathway. This mechanism diagram summarizes the core findings of this study and provides an intuitive framework for subsequent discussions on metabolic intervention strategies.

**Figure 10:**
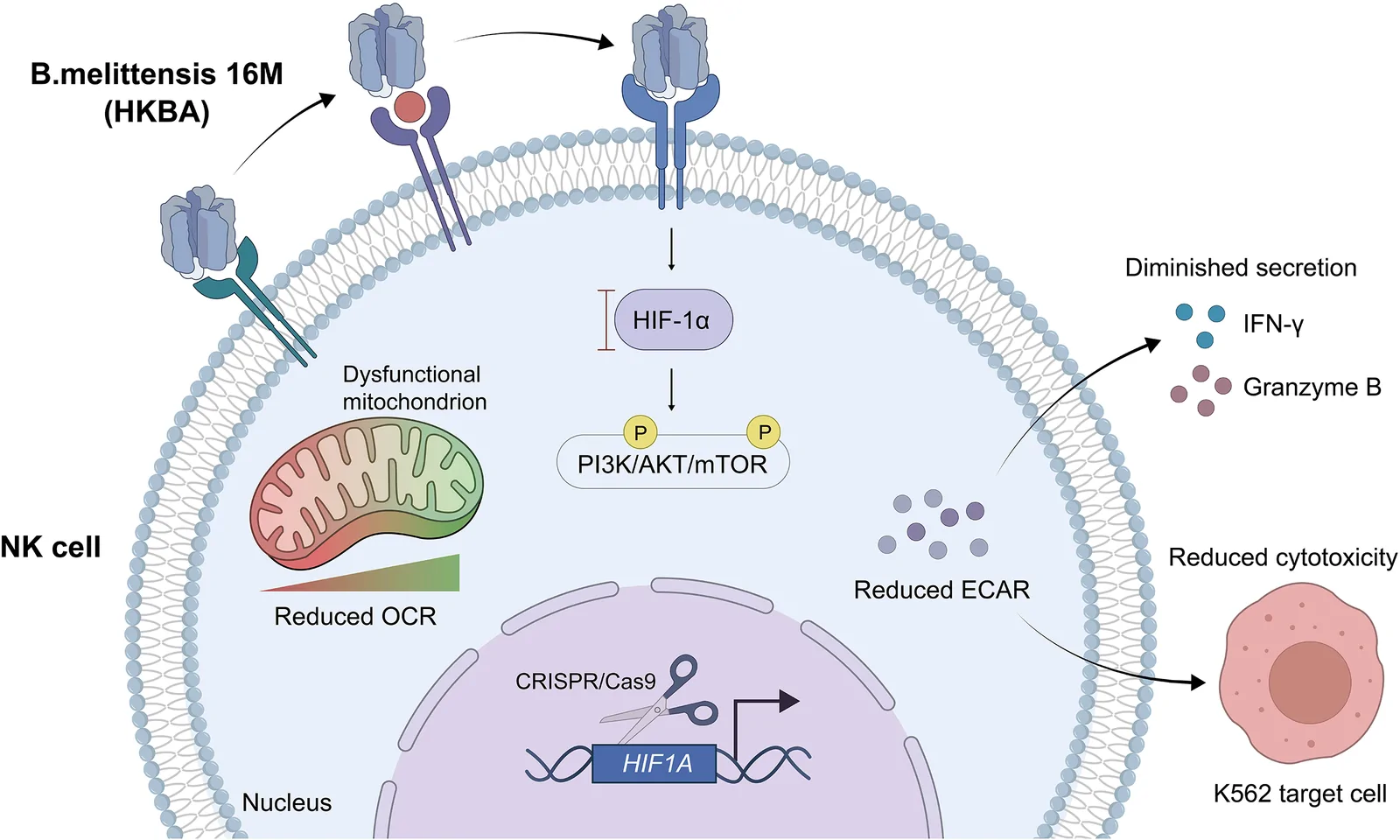
Schematic model of the mechanism by which Brucella induces NK cell metabolic reprogramming and functional suppression through the HKBA–HIF-1α–mTOR axis.

## 4 Discussion

In this study, we systematically characterized, for the first time, the functional inhibition and metabolic distress phenotypes of NK cells in brucellar arthritis, and revealed the underlying regulatory mechanism of the HIF-1α–mTOR signaling axis. Our major findings include (1): NK cells are enriched in the synovium of brucellar arthritis patients, especially in the chronic phase, but exhibit impaired oxidative phosphorylation (OXPHOS), and peripheral blood NK cells show significantly reduced effector functions (2); this functional inhibition is closely associated with aberrant HIF-1α overexpression and persistent suppression of mTOR pathway activity in NK cells (3); *in vitro* experiments confirmed that HKBA upregulates HIF-1α and inhibits mTOR signaling in NK-92 cells, leading to decreased mitochondrial membrane potential and reduced glycolytic capacity (4); CRISPR/Cas9-mediated HIF-1α knockout significantly restores mTOR activity, mitochondrial respiration, glycolytic function, and cytotoxic capacity of NK cells, while the mTOR inhibitor rapamycin completely blocks this restorative effect, confirming that HIF-1α drives NK cell metabolic reprogramming primarily through mTOR pathway inhibition (44).

The role of NK cells in anti-Brucella infection has been preliminarily recognized, but previous studies have mostly focused on changes in peripheral blood cell counts or cytokine secretion capacity during the acute phase (45), with little attention to the joint local microenvironment or the mechanisms of functional suppression in the chronic phase. Our single-cell transcriptomic data reveal for the first time that although NK cells are recruited to the inflammatory site in the synovium of chronic brucellar arthritis patients, their mTOR signaling pathway and OXPHOS-related gene sets are significantly downregulated, with no compensatory upregulation of glycolytic genes, forming a state of “metabolic rigidity.” This phenomenon is similar to the metabolic distress of NK cells in the tumor microenvironment (37, 46), but the metabolic reprogramming driven by persistent antigen stimulation in the context of infection may involve different upstream signals (47). We further confirmed that both HIF-1α protein and mRNA are significantly elevated in peripheral blood NK cells of chronic-phase patients, accompanied by reduced levels of p-PI3K, p-AKT, and p-mTOR, suggesting that Brucella may actively suppress NK cell metabolic checkpoints in a HIF-1α-dependent manner (48).

Mechanistically, HKBA may stabilize HIF-1 α under normoxic conditions through the TLR2/TLR4 mediated signaling pathway (49). Bacterial PAMPs such as lipoproteins and LPS can produce reactive oxygen species (ROS) and promote succinic acid accumulation, both of which can inhibit prolyl hydroxylase (PHD) - the key oxygen sensing enzyme that targets HIF-1 α for ubiquitination degradation (42, 50). This PAMP driven PHD inhibition results in the stable expression and translocation of HIF-1 α protein into the nucleus even in the absence of hypoxia. This mechanism has been reported in the study of bacterial lipopolysaccharides and other pathogenic components. This pathway provides a reasonable explanation for the upregulation of HIF-1 α observed in NK-92 cells under normoxic culture conditions after HKBA treatment.

HIF-1α, as a core transcription factor in hypoxia and inflammatory responses, exerts dual regulation on the mTOR pathway. In tumor models, HIF-1α suppresses mTORC1 through induction of REDD1 or activation of the TSC1/2 complex (51), leading to restricted effector functions of T cells and NK cells (52). Our study is the first to validate this mechanism in an intracellular bacterial arthritis model: HKBA treatment directly upregulates HIF-1α, accompanied by a comprehensive decline in mTOR pathway phosphorylation levels; HIF-1α knockout not only restores mTOR activity but also reverses the dual suppression of mitochondrial respiration and glycolysis induced by HKBA (53). Notably, Seahorse results showed that HIF-1α knockout restored both OXPHOS and glycolytic capacity, rather than merely shifting toward one pathway, indicating that in the context of Brucella infection, the metabolic defect in NK cells is a global energy supply disorder rather than a simple failure of metabolic switching. This finding aligns with the concept of “metabolic flexibility”: NK cells need to maintain both mitochondrial reserve and glycolytic capacity to respond to infectious stimuli, and aberrant accumulation of HIF-1α disrupts this flexibility (54).

The clinical significance of our study lies in proposing a novel therapeutic strategy targeting the metabolic axis. Current treatment for brucellar arthritis relies primarily on antibiotics, but the efficacy is limited in chronic-phase patients, and it cannot reverse established joint destruction. Restoring NK cell immune function holds promise for breaking Brucella immune evasion, but direct use of activating factors such as IL-2 may cause excessive inflammation (55). Our study suggests that inhibiting HIF-1α (e.g., using glycolysis inhibitors or HIF-1α-specific small molecules) or activating the mTOR pathway (e.g., using low-dose rapamycin analogs) may more “physiologically” restore NK cell energy metabolism, thereby enhancing pathogen clearance without exacerbating inflammation. Of course, such strategies require careful evaluation of their double-edged effects on local joint inflammation in animal models.

This study has certain limitations. Firstly, the number of rare subpopulations such as NK_CD56bri in the scRNA seq data of this study is extremely limited (only 7–20 cells per group). Although pathway scoring and correlation analysis based on this subpopulation reveal potential trends, their statistical robustness is insufficient, and the relevant conclusions should be considered as exploratory findings that need to be further validated through targeted sequencing after flow cytometry purification. And the patient sample size is relatively small (20 cases per group), and it is a single center study. The extrapolation of the conclusions still needs to be verified by a multi center cohort. Secondly, the HIF-1 α knockout NK-92 cell line used in this study was only derived from one CRISPR monoclonal antibody (1D9). Although we validated double allele deletion through three region PCR and confirmed homozygous linkage sequences through Sanger sequencing, we acknowledge that the lack of independent second clones or gene complementation/rescue experiments is a limitation. The possibility of clone specific off target effects cannot be completely ruled out, and should be considered when interpreting mechanism evidence. It is worth noting that our pharmacological rescue experiment using the mTOR inhibitor rapamycin (**Figure 9**) functionally confirmed that the phenotype recovery mediated by HIF-1 α knockout is achieved through the mTOR pathway, which to some extent compensates for this limitation. Establishing independent knockout clones and conducting rescue experiments for HIF-1 α overexpression in future research is crucial for further consolidating our conclusions. Thirdly, as a whole cell antigen model, HKBA contains various toxic components such as LPS and outer membrane proteins (56), which may affect NK cells through synergistic effects; Subsequently, live Brucella should be used for infection experiments to verify (57). We acknowledge that the use of HKBA has inherent limitations. As demonstrated by Harms et al. (23) in the SKG mouse model, heat inactivated Brucella cannot induce the significant arthritis observed in live bacterial infection, indicating that live bacterial infection and persistent bacterial presence are key factors for complete disease development in the body. In addition, the HKBA dose (MOI = 100) used in the *in vitro* experiments in this study represents a higher PAMP load, which may induce acute innate immune responses different from the intracellular infection properties of live bacteria - live bacteria can invade host cells, establish intracellular niches, persist, and dynamically interact with host pathways. Therefore, the simultaneous decrease in OCR and ECAR after HKBA treatment may reflect the widespread metabolic stress response after high-dose antigen stimulation, rather than the specific metabolic adaptation that occurs during effective intracellular Brucella infection. Therefore, our conclusion regarding the HIF-1 α/mTOR axis should be interpreted as a metabolic effect induced by Brucella antigens on NK cells, and caution should be exercised when extrapolating to live bacterial infections. We have identified this as a core limitation. However, as mentioned in the introduction, HKBA has a unique advantage in deciphering direct PAMP driven metabolic events triggered by Brucella antigens - which are associated with antigen rich microenvironments in the chronic phase of Brucella arthritis - while excluding confounding factors such as bacterial metabolic consumption and host active manipulation mediated by T4SS. Future research on the use of live Brucella under appropriate biosafety conditions is crucial for verifying whether the metabolic reprogramming observed in this study is reproduced in real intracellular infections. Fourthly, most of the mechanism experiments, including Seahorse metabolism analysis, JC-1 mitochondrial membrane potential detection, and transcriptome sequencing, were only conducted in the NK-92 cell line and were not validated in primary patient NK cells. Although NK-92 is a widely accepted and well identified NK cell biological model, primary NK cells may exhibit different metabolic responses due to their different activation states, tissue sources, and individual differences. This is the core limitation of the evidence for this mechanism, and we cannot assume that the metabolic phenotype observed in NK-92 cells is fully reproduced in primary NK cells. The study of using freshly isolated patient derived primary NK cells and *in vivo* animal models in the future is crucial for validating our findings. In addition, due to biosafety limitations and the current technical conditions of BSL-2 cell culture facilities, this study was unable to directly compare the metabolic reactions (OCR and ECAR) of live Brucella and heat inactivated Brucella in the same experimental system. This type of comparison is crucial for determining whether the HIF-1 α/mTOR related metabolic phenotype is specific to live bacterial intracellular infections or has reproducibility within them. We acknowledge this as a significant limitation and emphasize that the findings of this study should be validated in a live bacterial infection model before extrapolating to the background of intracellular Brucella infection. Fifth, this observational study did not systematically control the potential clinical confounding factors such as the history of previous antibiotic treatment, duration of disease, and complications (such as diabetes and hypertension). These factors may affect the phenotype, metabolic status, and functional response of NK cells, and should be considered when interpreting clinical data. In the future, larger and more rigorously controlled cohort studies are needed to control for these variables.

## 5 Conclusion

This study is the first to reveal a novel metabolic regulatory mechanism underlying NK cell functional suppression in brucellar arthritis (4). We demonstrate that Brucella, through its bacterial components (such as LPS, outer membrane proteins, etc.), induces aberrant HIF-1α overexpression in NK cells, which in turn inhibits the mTOR signaling pathway, leading to metabolic reprogramming characterized by impaired oxidative phosphorylation (OXPHOS) and rigid energy metabolism (42), ultimately resulting in significantly reduced effector functions and promoting the chronic progression of arthritis. Using HIF-1α gene knockout and mTOR inhibitor intervention, we have confirmed the central role of this signaling axis from both gain-of-function and loss-of-function perspectives. The findings of this study not only deepen our understanding of the immunopathological mechanisms of brucellar arthritis but also provide a theoretical basis for developing immunotherapeutic strategies that restore NK cell function by targeting the HIF-1α/mTOR metabolic axis.

## Data Availability

The datasets presented in this study can be found in online repositories. The names of the repository/repositories and accession number(s) can be found in the article/**Supplementary Material**.
