# Supplementary material for: Metabolic reprogramming of NK cells in chronic brucellar arthritis: role of the HIF-1α/mTOR axis
Source: Front Immunol. 2026 Oct 6;17:1961240. doi: 10.3389/fimmu.2026.1961240 (PMC13680154; doi:10.3389/fimmu.2026.1961240)
Supplement: Supplementary file 1 [file SupplementaryFile1.docx]

**A**

| **Marker** | **Region 1 (WT: 810 bp; heterozygous: 810 bp; homozygous: 0 bp)** | **Region 2 (WT: 896 bp; heterozygous: 896 bp; homozygous: 0 bp)** | **Region 3 (WT: 19491 bp; heterozygotes: 19491/~958 bp; homozygotes:~958 bp)** |
| --- | --- | --- | --- |
|  |  |  |  |

B

( A GRNA targeting sites and PCR identification regions on the wild-type hHIF1A allele.B Sanger sequencing peak map of monoclonal 1D9.)
